# Multidrug transporter MRP4/ABCC4 as a key determinant of pancreatic cancer aggressiveness

**DOI:** 10.1038/s41598-020-71181-w

**Published:** 2020-08-26

**Authors:** A. Sahores, A. Carozzo, M. May, N. Gómez, N. Di Siervi, M. De Sousa Serro, A. Yaneff, A. Rodríguez-González, M. Abba, C. Shayo, C. Davio

**Affiliations:** 1 Instituto de Investigaciones Farmacológicas (ININFA-UBA-CONICET), Facultad de Farmacia y Bioquímica, Universidad de Buenos Aires, Junín 956, C1113AAD Buenos Aires, Argentina; 2 Instituto de Biología y Medicina (IBYME-CONICET), Buenos Aires, Argentina; 3 Centro de Investigaciones Inmunológicas Básicas y Aplicadas, Facultad de Ciencias Médicas, Universidad Nacional de La Plata, Buenos Aires, Argentina

**Keywords:** Cancer, Gastrointestinal cancer

## Abstract

Recent findings show that MRP4 is critical for pancreatic ductal adenocarcinoma (PDAC) cell proliferation. Nevertheless, the significance of MRP4 protein levels and function in PDAC progression is still unclear. The aim of this study was to determine the role of MRP4 in PDAC tumor aggressiveness. Bioinformatic studies revealed that PDAC samples show higher MRP4 transcript levels compared to normal adjacent pancreatic tissue and circulating tumor cells express higher levels of MRP4 than primary tumors. Also, high levels of MRP4 are typical of high-grade PDAC cell lines and associate with an epithelial-mesenchymal phenotype. Moreover, PDAC patients with high levels of MRP4 depict dysregulation of pathways associated with migration, chemotaxis and cell adhesion. Silencing MRP4 in PANC1 cells reduced tumorigenicity and tumor growth and impaired cell migration. Transcriptomic analysis revealed that MRP4 silencing alters PANC1 gene expression, mainly dysregulating pathways related to cell-to-cell interactions and focal adhesion. Contrarily, MRP4 overexpression significantly increased BxPC-3 growth rate, produced a switch in the expression of EMT markers, and enhanced experimental metastatic incidence. Altogether, our results indicate that MRP4 is associated with a more aggressive phenotype in PDAC, boosting pancreatic tumorigenesis and metastatic capacity, which could finally determine a fast tumor progression in PDAC patients.

## Introduction

Pancreatic ductal adenocarcinoma (PDAC) is one of the most lethal human malignancies, due to its late diagnosis, inherent resistance to treatment and early dissemination^1^. This type of tumor is expected to become the second leading cause of cancer mortality by the year 2030 and has limited therapeutic options^2^. Even after the development of new targeted agents and the use of multiple therapeutic combinations, there is no clear benefit for this disease. Thus, there is an unmet need to propose novel strategies to target PDAC and improve its prognosis.

Multidrug Resistance-Associated Protein 4 (MRP4/ABCC4) is a member of the ATP-binding cassette (ABC) protein family that act mainly as molecular transporters and as such, are key participants in the emergence of multidrug resistance in cancer^3^. Also, MRP4 endogenous substrates may participate in signaling transduction, cell proliferation, metabolism, survival, apoptosis, senescence, exocytosis and endocytosis^4–6^. In addition, MRP4 actively affects tumorigenesis and has been associated with progression in numerous types of cancers, including blood, brain, colon, liver, lung, and prostate^7^ but has only been briefly described in pancreatic cancer^8^.

We have recently proved that MRP4 is a key participant in PDAC cell proliferation, mainly by regulating cAMP efflux^9^. We described that MRP4 mRNA and protein levels inversely correlate with the degree of differentiation of pancreatic cancer cell lines and that PDAC patients with high expression of MRP4 tend to have a shorter overall survival compared to patients with low MRP4 expression. These findings suggest that MRP4 has tumor promoting functions and that high levels of MRP4 expressed selectively in human pancreatic tumor tissues may contribute to the development of high-risk pancreatic cancer.

Using available whole genome expression datasets, in this study we sought to validate our hypothesis that MRP4 is associated with a more aggressive phenotype in PDAC. We also explore whether MRP4 influences PDAC tumor growth and its ability to spread, including cell migration and in vivo tumor dissemination studies in pancreatic cancer models and immunosuppressed mice. Our findings will help us determine the role of MRP4 in PDAC tumor biology and set the basis to further validate this transporter as a potential prognosis biomarker and/or therapeutic target for PDAC.

## Results

### Bioinformatic analysis of MRP4 differential expression in PDAC samples, PDAC cell lines and CTC from PDAC patients

**Figure 1:**
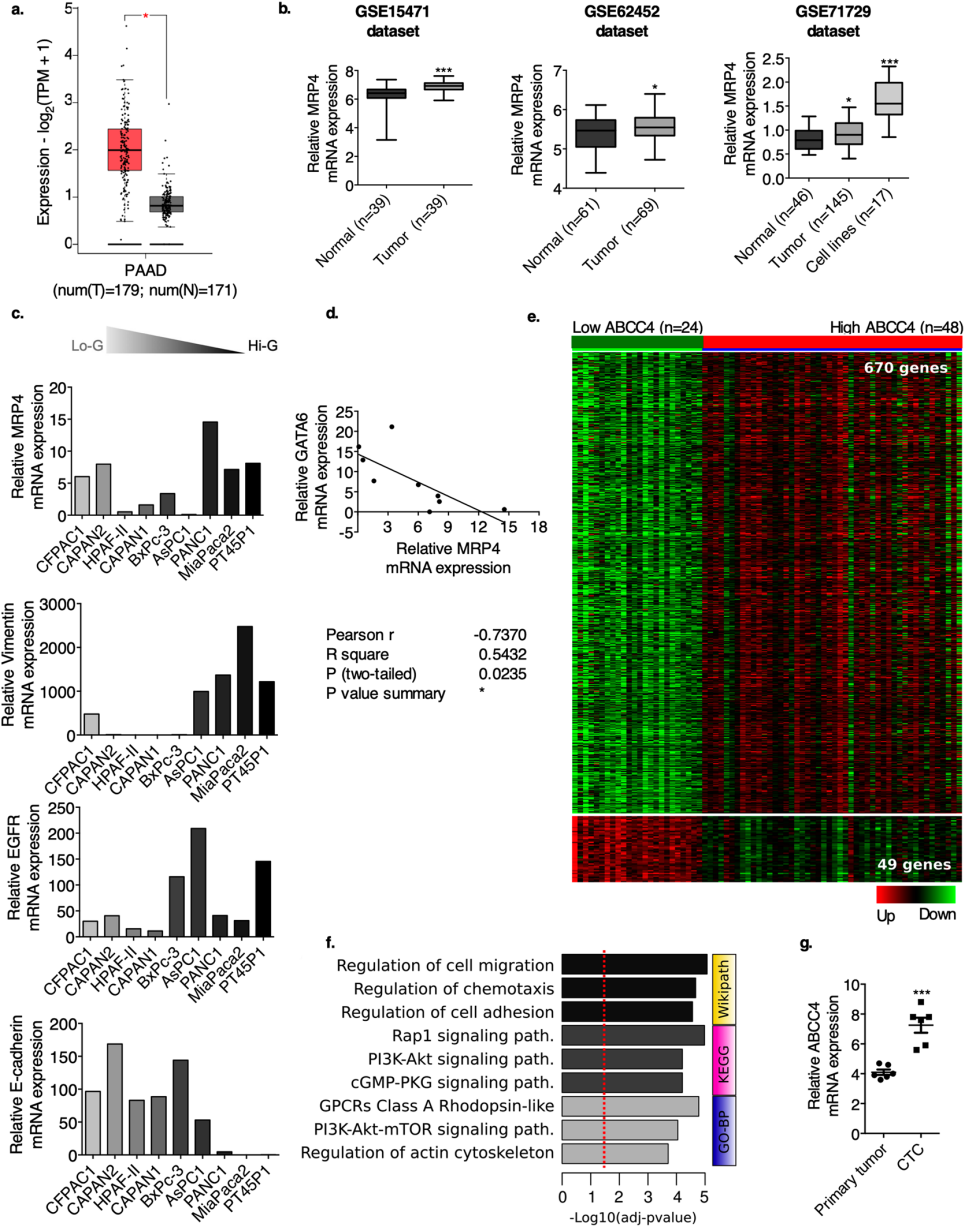
Bioinformatic analysis of MRP4 differential expression in PDAC samples, normal/adjacent pancreatic tissue, PDAC cells lines and circulating tumor cells from PDAC patients. (**a**) Differential expression of MRP4 according to the Gene Expression Profiling Interactive Analysis (GEPIA) database using TCGA PAAD tumor data and matched data of normal tissue from TCGA. (**b**) Comparative analysis of MRP4 mRNA expression from three different gene expression datasets comprising pancreatic normal tissue and their paired PDAC samples (GSE15471 and GSE62452), as well as PDAC cell lines (GSE71729). (**c**) MRP4, vimentin, E-cadherin, and EGFR transcript levels in a panel of 9 pancreatic cancer cell lines systematically arranged from the less aggressive (Lo-G) to the more aggressive (Hi-G) phenotypes (GSE64557). (**d**) Inverse correlation between MRP4 and GATA6 expression levels in the 9 pancreatic cancer cell lines from the beforementioned dataset. (**e**) Heatmap showing differentially expressed genes in MRP4 LE and HE PDAC samples performed with MultiExperiment Viewer (MeV). Differentially expressed genes were identified based on a log2(fold change) ≥ 1 and an FDR ≤ 0.05. Hierarchical clustering was based on Pearson correlation coefficients. (**f**) Functional enrichment analysis of differentially expressed genes showing pathways associated with MRP4 expression (p-adjusted < 0.05). See Supplementary Table S1. (**g**) Negative depletion fluorescence activated cell sorting (FACS) was used to enrich for CTC from the blood of 6 patients who underwent surgery for PDAC (GSE18670). Positive 7-amino-actinomycin D viability and negative CD45 and CD34 cells were identified as CTC. We considered mRNA levels of ABCC4 1552918 isoform an indicator of MRP4 expression levels. Statistics: *t*-test ***p < 0.001 and *p < 0.05.

To investigate MRP4 expression in human pancreatic cancer, we performed a differential expression analysis comparing PDAC and normal pancreatic tissue using several online datasets. GEPIA analysis showed a significant MRP4 upregulation in tumor samples compared to normal tissues (Fig. 1a). Similarly, the expression data from three different datasets from the Gene Expression Omnibus (GEO: GSE15471, GSE62452, and GSE71729) revealed higher MRP4 transcript levels in PDAC specimens relative to matching normal samples (*p* < 0.001 and *p* < 0.05; Fig. 1b). Bioinformatic analysis of GSE71729 dataset also evidenced that MRP4 is upregulated in 17 established pancreatic cancer cell lines compared to tumor and peritumor samples (*p* < 0.001), suggesting that elevated expression of MRP4 is associated with the process of carcinogenesis and may confer an adaptive phenotype to cells growing in culture.

In agreement with recent studies from our laboratory^9^, we confirmed that MRP4 transcript levels inversely correlate with PDAC cell line differentiation grade (GSE64557 dataset^10^; n = 9; Fig. 1c). Low-grade tumors tend to have better prognosis and highly express adhesion and epithelial genes, while high-grade tumors are associated with a worse prognosis and show high expression of mesenchymal genes^11^. In line with this, we further explored vimentin and E-cadherin expression in the same panel of PDAC cell lines, as both proteins are associated with the process of epithelial-mesenchymal transition (EMT). The bioinformatic analysis evidenced that high-grade PDAC cell lines express high levels of vimentin and low levels of E-cadherin, whereas the opposite is observed in low-grade PDAC cell lines. In addition, high-grade PDAC cell lines depict EGFR upregulation, which has been associated with advanced stage, metastatic disease, and poor differentiation and survival in pancreatic adenocarcinoma^12,13^. Interestingly, a significant inverse correlation was observed between MRP4 and GATA6 expression levels (*p* < 0.05; Fig. 1d), which has been recently described to inhibit EMT and tumor dissemination in pancreatic cancer^14^. Collectively, our observations indicate that high MRP4 transcript levels could be linked to the maintenance of a mesenchymal phenotype and determine a poorer outcome in patients with pancreatic cancer.

As reported in Carozzo-Yaneff et al., we used the gene expression profile obtained from TCGA-PAAD RNAseq dataset (n = 178; UCSC Xena), and classified it into two groups of patients according to low (LE; 24/178) or high (HE; 48/178) MRP4 mRNA levels^9^. Taking in consideration we had already described that MRP4 HE patients tend to have shorter overall survivals compared to MRP4 LE patients, we further assessed the differential gene expression between both groups. We now identified 720 genes differentially expressed between HE and LE MRP4 carcinomas (FDR < 0.05; Fold changes > 2; Fig. 1e). Ninety three % of the identified transcripts (671/720) were upregulated in HE tumors, while only 6.8% (49/720) were downregulated genes (Supplementary Table S1). Interestingly, functional enrichment analysis of dysregulated transcripts revealed specific bioprocesses characteristic of MRP4 HE tumors; strongly associated with the regulation of cell migration (*p* = 3.65e−8), chemotaxis (*p* = 1.21e−7), and cell adhesion (*p* = 1.98e−7), consistent with a mesenchymal and aggressive phenotype. Furthermore, we also identified a high association between GPCR (*p* = 6.45e−7), PI3K-Akt-mTOR (*p* = 8.29e−7), and Rap1 (*p* = 4.66e−8) signaling pathways with MRP4 expression in PDAC cases (Fig. 1f). The dysregulation of these genes and processes could result in the pathogenesis or progression of pancreatic cancer.

Circulating tumor cells (CTC) are key for metastatic spreading as they detach from the primary tumor mass and survive in distant organs. In this sense, we took advantage of the public GSE18670 whole genome microarray, consisting of CTC isolated from the blood of six patients who underwent surgery for PDAC^15^. We observed that MRP4 transcript levels are upregulated in CTC compared to primary tumor cells of PDAC patients (*p* < 0.001; Fig. 1g). This observation suggests that MRP4 upregulation may confer tumor cells a greater ability to survive in circulation, enhancing their migratory and metastatic phenotype.

### Effect of MRP4 silencing in PANC1 xenograft growth and cell migration

With the intention of evaluating the biological effect of MRP4 modulation upon tumor aggressiveness, we carefully selected two cell lines with dissimilar MRP4 protein levels and differentiation grade: PANC1 cells depict high levels of MRP4 and are considered poorly differentiated, whereas the moderately differentiated BxPC-3 cells^16^ express intermediate MRP4 levels. We have recently established that MRP4 silenced PANC1 clones exhibit a slower proliferation curve compared to scramble cells and that BxPC-3 MRP4-overexpressing clones proliferate more than mock cells^9^.

**Figure 2:**
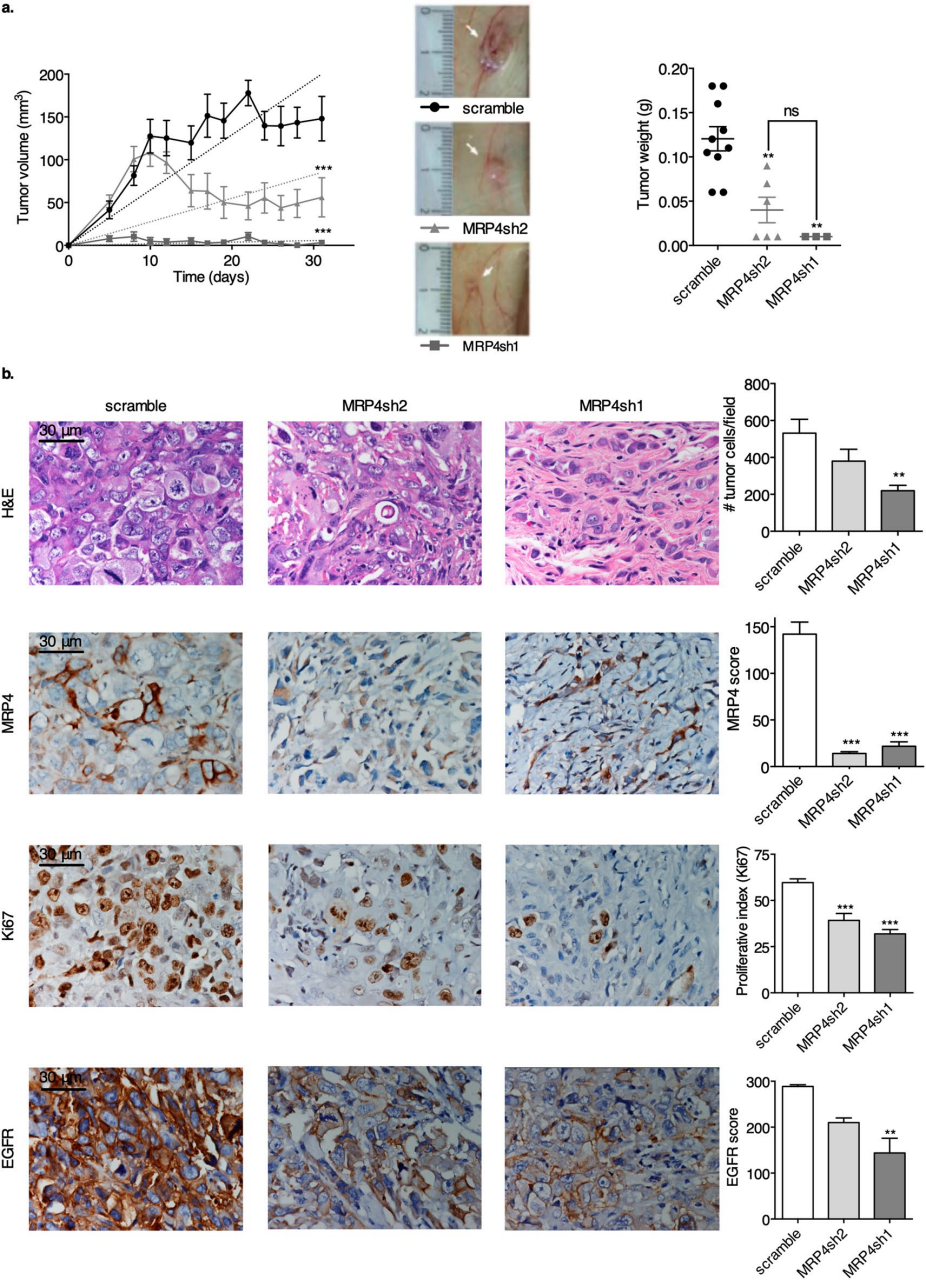
MRP4 silencing reduces tumor growth and tumorigenicity in PANC1 xenografts. Swiss nude mice were subcutaneously injected either with PANC1-MRP4sh2, PANC1-MRP4sh1 clones or PANC1-scramble as a control. Each group contained 8–11 animals and the data shown represents measurements of one of two experiments. (**a**; left) Tumors were measured with a caliper three times a week for 32 days post-inoculation and tumor volume was calculated as described in “Materials and Methods”. (**a**; middle) Representative pictures of the dissected xenografts of each group at the end of the experiment. (**a**; right) Tumor weight was measured with a scale after dissection. (**b**) PANC1-scramble, PANC1-MRP4sh2 and PANC1-MRP4sh1 xenografts were processed for histological (H&E staining), MRP4, Ki67, and EGFR immunostaining evaluation. Nuclei were counterstained with hematoxylin. Representative images are shown. Cell number per field, proliferative index, MRP4 and EGFR scores were determined by H&E, Ki67, MRP4, and EGFR staining respectively, and are shown as bar plots (right). Data is shown as mean ± SEM. Statistics: Linear regression and one-way ANOVA followed by Tukey’s *t*-test. ***p < 0.001 and **p < 0.01.

In order to assess the relevance of MRP4 inhibition on tumor aggressiveness, we first inoculated nude mice with scramble cells and the two MRP4-silenced PANC1 clones (MRP4sh1 and sh2), and then evaluated tumor incidence and growth. As described in our recent publication, MRP4sh1 cells express lower MRP4 levels than MRP4sh2 cells (49% and 69% compared to scramble cells, respectively)^9^. Silencing MRP4 strongly reduced tumor incidence: 91% (10/11) for scramble cells, 55% (6/11) for MRP4sh2 cells and 27% (3/11) for MRP4sh1 cells (Fig. 2a). Linear regression of the tumor growth curves evidenced that all silenced PANC1 xenografts grew significantly slower than the scramble cells (*p* < 0.001), and that MRP4sh1 tumors grew even less than MRP4sh2 tumors. Moreover, MRP4sh xenografts which did grow, displayed smaller tumor masses compared to the scramble cell line (*p* < 0.001). Our results reveal that silencing MRP4 significantly disrupts PANC1 tumor formation and growing capacity.

Histological analysis revealed that all xenografts displayed a non-differentiated pattern, as cells do not form any kind of glandular structure (Fig. 2b). Tumors are composed by solid tumor sheets of atypical cells with very little stromal septa, like PANC1 parental tumors^17^. The xenografts show moderate amounts of blood vessels, perineural invasion and infiltrating margins. In accordance with the observed growth rate, silenced xenografts display a smaller number of viable epithelial neoplastic cells (quantified as cell number per field; *p* < 0.01) and an increased amount of stromal component, both evidenced by hematoxylin and eosin (H&E) and Pan-CK staining (data not shown). Immunostaining for MRP4 confirmed that MRP4sh1 and MRP4sh2 xenografts are indeed silenced, as they display almost no MRP4 staining compared to scramble tumors (*p* < 0.001). Proliferation rate, determined by Ki67 immunostaining, and EGFR score were consistent with MRP4 expression levels, showing a diminished proliferation index and EGFR immunostaining in silenced tumors compared to scramble tumors (*p* < 0.001 and *p* < 0.01, respectively; Fig. 2b).

**Figure 3:**
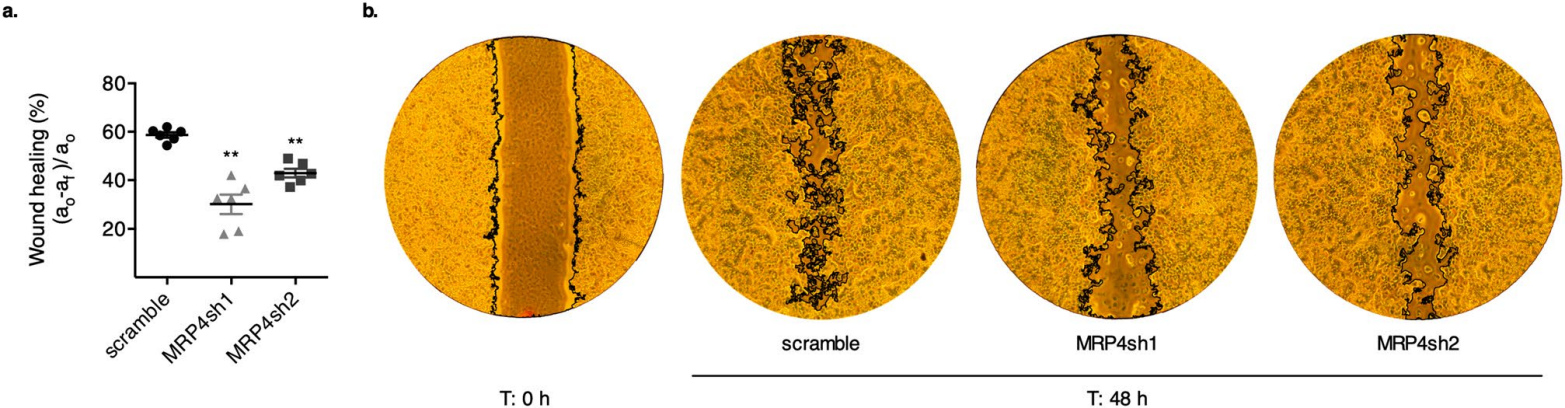
MRP4 silencing impairs cell migration in PANC1 clones. (**a**) PANC1-scramble, PANC1-MRP4sh1 and PANC1-MRP4sh2 cell migration was assessed by closure of the wound. The area of the scratch was measured at T:0 h and T:48 h in all cell lines, and % reduction of initial scratch area was compared as described in “Materials and methods”. Data is shown as mean ± SEM of six measurements and the experiment was performed three times. (**b**) Representative images of a wound at starting point (T: 0 h), and PANC1-scramble, PANC1-MRP4sh1 and PANC1-MRP4sh2 cell migration into the wound at 48 h post scratch are shown. Wound areas are delimited in black. Statistics: One-way ANOVA followed by Tukey’s *t*-test. **p < 0.01.

Further, we assessed whether MRP4 silencing also affected PANC1 migratory ability in culture. The wound-healing assay showed that MRP4 downregulation significantly impaired the migratory phenotype of PANC1 cells (*p* < 0.05; Fig. 3). These results suggest that MRP4 determines PDAC aggressiveness per se and that its expression levels may correlate with PDAC metastatic potential.

### Impact of MRP4 silencing on cell processes that may determine PDAC level of aggressiveness

**Figure 4:**
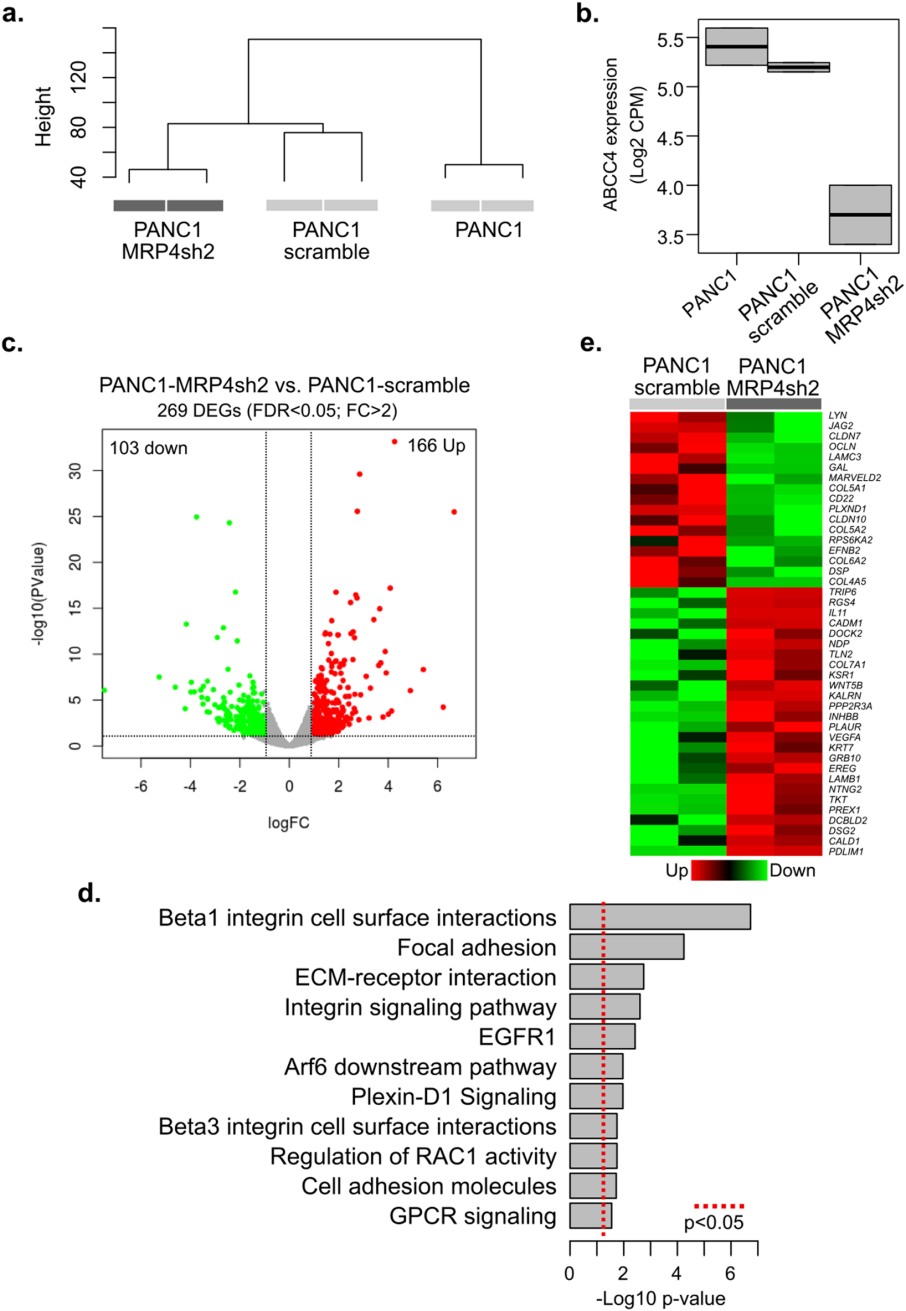
MRP4 silencing reprograms gene expression, mainly dysregulating pathways related to cell surface interactions, focal adhesion, and extracellular matrix remodeling. (**a**) Unsupervised clustering of PANC1 cell lines based on transcriptomic profiles. (**b**) Box plot of MRP4 (ABCC4) mRNA expression between PANC1 cell lines, showing MRP4 is underexpressed in MRP4sh2 compared to control (PANC1 and PANC1-scramble) cells. (**c**) Volcano plot representing the significance (− Log10 p-value) and magnitude of transcript change (Log2FC) in scramble and MRP4sh2 cells. The red dots represent significantly upregulated transcripts and the green dots represent downregulated transcripts upon MRP4 silencing (FDR < 0.05; Fold Changes > 2). (**d**) Functional enrichment analysis were performed using the Enrichr resource (https://amp.pharm.mssm.edu/Enrichr) and InnateDB (https://www.innatedb.com) based on the list of dysregulated transcripts between scramble and MRP4sh2 cells (FDR < 0.05; Fold changes > 2). See Supplementary Table S2. (**e**) Heatmap showing differentially expressed genes related to the enriched signaling pathways. Hierarchical clustering was based on Pearson correlation coefficients.

To investigate how MRP4 may determine the level of aggressiveness in PDAC, we performed an RNAseq analysis of PANC1, PANC1-scramble and PANC1-MPR4sh2 transcriptomes. Unsupervised analysis revealed that all three cell lines are readily separated from each other and that biological replicates cluster together (Fig. 4a). As expected, MRP4sh2 cells display downregulation of the MRP4 gene (ABCC4) compared to control (scramble and parental PANC1) cells (Fig. 4b). By performing a differential expression analysis comparing MRP4sh2 and scramble cells, we were able to identify a gene set of 269 (166 upregulated and 103 downregulated; FDR < 0.05; Fold changes > 2) transcripts that showed significant expression changes when MRP4 was suppressed (Fig. 4c, Supplementary Table S2).

Interestingly, functional enrichment analysis revealed that cell surface interactions (*p* = 1.88e−7), focal adhesion (*p* = 5.70e−5), and extracellular matrix interactions (*p* = 1.79e−3), appear to be among the most dysregulated processes in MRP4sh2 cells (*p* < 0.05; Fig. 4d,e). This could compromise the migratory and invasive ability of PANC1 cells. Differential expression of genes related to the Arf6, (*p* = 1.07e−2), Plexin-D1 (*p* = 1.07e−2) and Wnt (WNT5B: *p* = 5.14e−10, NRARP: *p* = 1.59e−6, and PRICKLE2: *p* = 4.73e−9, among other transcripts) cascades may also affect cell proliferation, differentiation and invasion, all processes associated with EMT and CTC phenotypes. Interestingly, IGFBP2, described as a pivotal regulator of an EMT axis in PDAC^18^ is downregulated in MRP4sh2 cells compared to scramble cells (*p* = 6.03e−5). Notably, IGFBP2 promotes invasion and metastasis of PDAC cells through NF-κB pathway, which is also altered in MRP4-silenced cells (Supplementary Table S2). In a similar way, the transcription factor GATA5 is also underexpressed (*p* = 3.04e−5; Supplementary Table S3, showing the top 10 down- and upregulated transcripts) and, as a key enhancer of histone marks associated with PDAC progression and dissemination in vivo^19^, may contribute to the resulting less aggressive phenotype of MRP4sh2 cells.

In coherence with data illustrated in Fig. 1, modulation of MRP4 levels also reverberates in GPCR signaling pathways (*p* = 2.80e−2). The GPCR-associated dysregulated genes in MRP4sh2 cells include: EREG, GAL, IL11, INHBB, JAG2, NDP, RGS4, VEGFA and WNT5B, among others (Supplementary Table S2). Although PI3K-Akt signaling pathway does not emerge as one of the main altered pathways upon MRP4 suppression, several genes related to this cascade are significantly modulated (Down: PPP2R3, VEGFA, EREG, LAMB1, and Up: LAMC3, COL6A2, COL4A5; Fig. 4e).

Altogether, these findings indicate that MRP4 silencing alters gene expression which may explain the slow proliferation and poor migratory capacity of MRP4sh cells. The data also highlights many of the signaling pathways which are dysregulated in PDAC tumor samples with dissimilar levels of MRP4 and points to an association between low levels of the transporter and a good prognosis in pancreatic cancer.

### Effect of MRP4 overexpression in BxPC-3 xenograft growth and experimental metastasis

**Figure 5:**
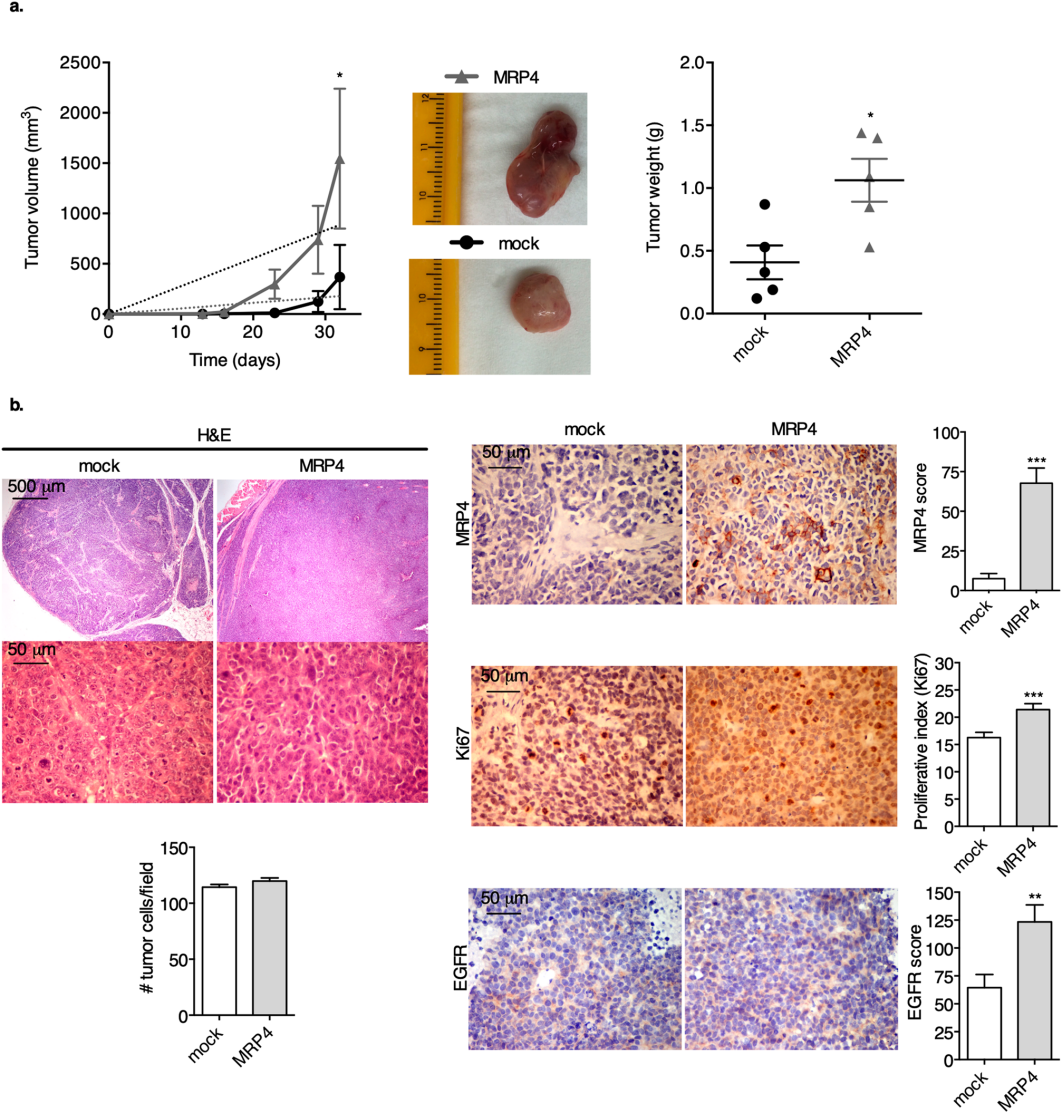
MRP4 overexpression increases tumor growth in BxPC-3 xenografts. NSG mice were subcutaneously injected with BxPC-3-MRP4 or BxPC-3-mock as a control. Each group contained 5 animals and the data shows measurements of one of two experiments. (**a**; left) Tumors were measured with a caliper three times a week for 32 days post-inoculation and tumor volume was calculated as described in “Materials and methods”. (**a**; middle) Representative pictures of the dissected xenografts of each group at the end of the experiment. (**a**; right) Tumor weight was measured with a scale after tumor dissection. (**b**) BxPC-3-mock and BxPC-3-MRP4 xenografts were processed for histological (H&E staining), MRP4, Ki67, and EGFR immunostaining evaluation. Nuclei were counterstained with hematoxylin. Representative images are shown. Cell number per field, proliferative index, MRP4 and EGFR scores were determined by H&E, Ki67, MRP4, and EGFR staining respectively, and are shown as bar plots (right). Data is shown as mean ± SEM. Statistics: Linear regression and Student´s *t*-test. ***p < 0.001, **p < 0.01, and *p < 0.05.

We then analyzed the xenograft growth curves of BxPC-3 clones in NSG mice and observed that MRP4 overexpression increases tumor burden, as BxPC-3-MRP4 xenografts display higher growth slopes and are heavier than mock tumors (Fig. 5a). In agreement with previous histological reports^20^, inoculated BxPC-3 clones form moderately differentiated adenocarcinomas, with traces of mucin, thin stromal tracts and are more comparable to pancreatic normal tissue than PANC1 xenografts (Fig. 5b). However, MRP4-overexpressing xenografts are less differentiated than mock tumors, as they reveal areas of solid tumor parenchyma with numerous mitotic and apoptotic figures. Immunostaining confirmed that these xenografts truly show elevated levels of MRP4 and an increased proliferative rate evidenced by intense MRP4 and Ki67 staining, respectively (*p* < 0.001). Regarding EGFR expression and localization, we found that MRP4-overexpressing tumors display a higher EGFR score compared to mock tumors (*p* < 0.01). EGFR upregulation upon MRP4 overexpression further sustains that MRP4 is associated with a poor prognosis, disease progression and higher aggressiveness in PDAC. Altogether, our findings support the hypothesis that MRP4 acts as a tumor booster and inspire the idea of targeting MRP4 as a novel therapy in pancreatic cancer.

**Figure 6:**
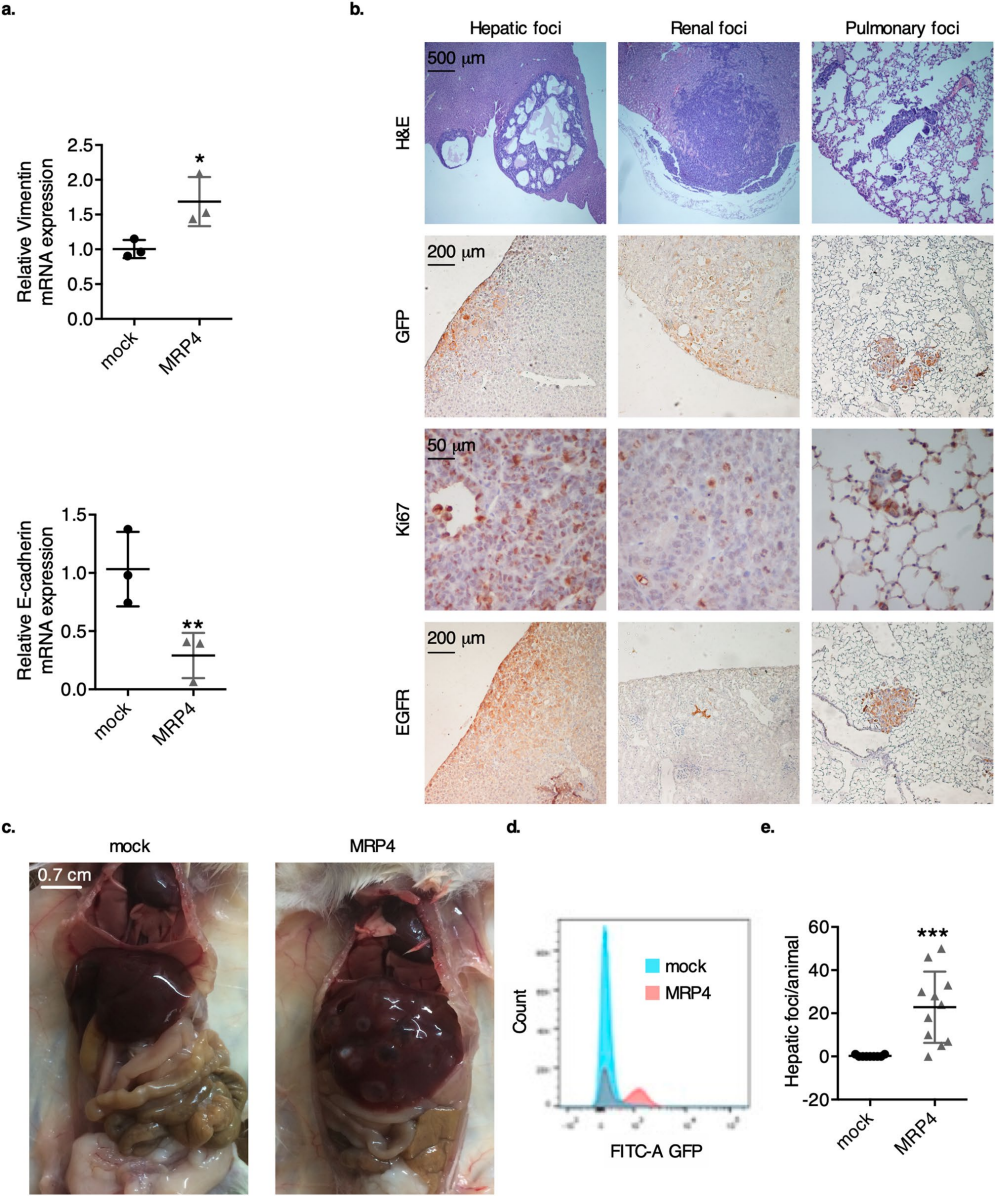
Effect of MRP4 overexpression upon BxPC-3 experimental metastasis. (**a**) Vimentin and E-cadherin (CDH1) transcript levels in BxPC-3-mock and BxPC-3-MRP4 cells, determined by qPCR as described in “Materials and methods”. Data is shown as mean ± SD of three measurements and the experiment was performed three times. (**b**) H&E staining, GFP, Ki67, and EGFR immunostaining of hepatic, renal, and pulmonary metastatic foci from BxPC-3-MRP4 inoculated mice. Nuclei were counterstained with hematoxylin. Representative images are shown. (**c**) Representative liver macro-metastases at the end of the experiment. (**d**) Representative comparison of GFP expression in liver homogenates, determined by FAC. (**e**) Number of hepatic metastatic foci per animal in each experimental group. Data is shown as mean ± SEM. Statistics: Student’s *t*-test. ***p < 0.001, **p < 0.01, and *p < 0.05.

To determine whether MRP4 modulation affects the expression of EMT markers in PDAC, we evaluated vimentin and E-cadherin (CDH1) mRNA levels by qPCR in BxPC-3 clones. BxPC-3-MRP4 cells express significant higher levels of vimentin and lower levels of E-cadherin compared to mock cells (Fig. 6a). We then investigated if MRP4 overexpression alters tumor spreading in vivo. BxPC-3-mock and BxPC-3-MRP4 cells both expressing GFP were inoculated into the tail vein of NSG mice. Four weeks later, the animals were humanely sacrificed, and the liver, kidneys and lungs were harvested to survey for CTC or metastatic foci. As shown in Table 1, incidence of metastases was significantly higher in the mice inoculated with MRP4-overexpressing cells compared to the mock group. Moreover, our data show that MRP4 overexpression augments metastatic incidence, especially to the liver, as all animals inoculated with BxPC-3-MRP4 cells exhibited hepatic macro-metastases at the end of the experiment (Table 1, Fig. 6b).

**Table 1:** Experimental metastasis is higher in mice inoculated with BxPC-3-MRP4 cells compared to the mock group.

	Hepatic foci	Renal foci	Pulmonary foci
BxPC-3-mock	20% (2/10)	10% (1/10)	30% (3/10)
BxPC-3-MRP4	100% (11/11)***	72.7% (8/11)**	81.8% (9/11)*

Incidence of localized metastases in each experimental group. Statistics: Chi-square and Fisher exact test to analyze metastatic incidence among experimental groups. ***p < 0.001, **p < 0.01, and *p < 0.05.

H&E staining and GFP immunostaining of liver, kidney and lung sections further verified the metastatic potential of BxPC-3-MRP4 cells and allowed the confirmation of small micro-metastases and CTC in the lungs (Fig. 6b; top). Specific immunostaining revealed that all metastatic foci were highly proliferative (Ki67) and continued expressing EGFR (Fig. 6b; bottom) as the parental xenografts displayed in Fig. 5b. Liver macro-metastases were numerous and evident at the end of the experiment (Fig. 6c) and FAC confirmed the presence of GFP + cells only in liver homogenates of BxPC-3-MRP4 inoculated mice (Fig. 6d). In fact, the BxPC-3-MRP4 group showed higher amount of hepatic metastatic foci per animal compared to the mock group (*p* < 0.001; Fig. 6e).

Endogenous GFP expression of BxPC-3 inoculated clones also allowed the visualization of renal and pulmonary experimental metastases in cryostat sections of the respective organ tissues (Supplementary Fig. S1). Regarding renal colonization, mice inoculated with BxPC-3-MRP4 cells depicted higher amount of individual metastatic foci compared to the mock group (*p* < 0.01). As evidenced by the Bouin’s fluid fixation, lungs of both experimental groups showed numerous miniature pulmonary metastatic foci, which were difficult to quantify with the naked eye or even under a light microscope. FAC analysis confirmed that the % of GFP+ fluorescent cells in lung homogenates from the BxPC-3-MRP4 group was significantly higher than that of the mock group (*p* < 0.01). Altogether, our findings indicate that MRP4 overexpression enhances colonization of pancreatic cancer cells to the liver, kidneys, and lungs in NSG mice.

## Discussion

In the present study we show that MRP4 is significantly upregulated in pancreatic cancer, PDAC cell lines and CTC from PDAC patients. MRP4 transcript levels inversely correlate with PDAC cell line differentiation grade, associate with an EMT signature, and determine PDAC tumor growth, migration and distant organ dissemination. Although more work is needed to validate our findings in PDAC samples, our results are the foundation to understand MRP4 association with PDAC aggressive behavior.

In our first approach, we were able to validate our previous results and test our hypothesis in an extensive PDAC cohort, using open access platforms. Our findings agree with those of Zhang et al., who examined by immunohistochemical staining 36 paired PDAC samples and reported that MRP4 protein levels are consistently augmented in pancreatic tumor samples compared to peritumor tissues^8^. We now analyzed four different gene expression datasets, comprising more than 300 primary pancreatic tumors and 200 normal/adjacent pancreatic samples, and established that MRP4 upregulation occurs at a genomic level in pancreatic tumor tissues and in PDAC cell lines as well. The fact that we established that high MRP4 levels: (a) are typical of high-grade pancreatic cancer cell lines, (b) co-express with mesenchymal markers, (c) cause differential expression of genes related with chemotaxis, migration and adhesion, and (d) are upregulated in CTC from PDAC patients, led us to propose that MRP4 is coupled to cancer development and plays a role in the maintenance of an aggressive phenotype. In line with this, several observations from clinical studies have shown that ABC transporters confer chemoresistance to cancer stem cells and are linked to the progression of malignant cancers^21^. In fact, MRP4 expression significantly associates with poor clinical outcomes in neuroblastoma and has been consequently validated as a powerful independent predictor of clinical outcome in patients with this tumor^22,23^. Altogether, our data suggest MRP4 could also be a potential prognostic biomarker in PDAC patients. Studies currently in progress, involving evaluation of MRP4 protein levels and subcellular localization by immunostaining in PDAC tissue microarrays will further validate MRP4 as an indicator predictor of clinical outcome in pancreatic cancer. For this, the relationship between MRP4 levels, tumor stage and patient outcome (chemo/radiotherapy response and overall survival) will be examined.

Few publications refer to the mechanisms responsible for induction of MRP4 expression in pancreatic cancer. MRP4 is upregulated in 5-FU-resistant pancreatic carcinoma cells^24^, although the underlying mechanism of this induction is still unclear. Long-term treatment with cAMP-enhancing agents augments MRP4 mRNA and protein levels in several cell types, involving the EPAC/MEK route rather than the PKA/CREB pathway^25^. Studies from our laboratory confirmed that, in pancreatic cancer cells, intracellular cAMP upregulates MRP4 through an Epac2/Rap1-mediated mechanism, whereas extracellular cAMP inhibits MRP4 promoter activity by a MEK/ERK-mediated pathway^26^. More recently, we described that an increase in intracellular cAMP levels induce MRP4 expression through the EPAC-PKA pathway in acute myeloid leukemia models^27^. MAPK signaling, through CREB1, NFκB, and EIF4E may also regulate MRP4 levels in human renal cells^28^*.* Moreover, Wnt/β-catenin signaling drives upregulation of MRP4 in human lung cancer cells, causing an increase in drug efflux and, thus, resistance to cisplatin^29^. Interestingly, many of the pathways and main actors associated with MRP4 transcriptomic regulation appeared to be dysregulated in our in silico analysis. Further research into the regulatory pathways that influence MRP4 expression specifically on pancreatic cancer is needed, as regulation of gene expression often depends on the cell system and context.

In this work, we selected PANC1 and BxPC-3 cell lines as models to study the role of MRP4 in pancreatic cancer progression. Phenotypically, both cell lines display differential expression levels of MRP4 and show distinct differentiation grades^30^. Genetically, PANC1 present mutations in KRAS, p53 and p16, while BxPC-3 present mutations in p53, p16 and Smad4, but depict a wild type KRAS^31,32^. We previously demonstrated that MRP4 silencing in PANC1 cells reduces the proliferation rate in culture^9^, and we now confirm a loss in tumorigenicity in vivo, as the incidence of palpable PANC1-MRP4sh xenografts significantly decreases compared to scramble xenografts. Conversely, MRP4 overexpression enhances BxPC-3 cell proliferation in culture compared to mock cells^9^, and we now verify that these xenografts grow more and have an elevated proliferative index in vivo, determined by Ki67 immunostaining. The evaluation of clinically relevant histopathological parameters further sustains that MRP4 is associated with a poor prognosis and higher aggressiveness in PDAC. Regardless the similarities and differences in the phenotype and genetic background of the PDAC cell lines used in our studies, these results validate our previous findings in an in vivo setting and indicate that MRP4 levels determine pancreatic tumor development, independently of KRAS status.

Additionally, the fact that in both cell models, MRP4 modulation alters EGFR score, which is associated with malignant transformation of pancreatic cancer and plays important roles in liver metastases and recurrence of human pancreatic cancer^12^, indicates that targeting MRP4 could eventually serve as a novel therapeutic strategy in PDAC.

Since our bioinformatic discoveries establish that MRP4 expression is associated with a mesenchymal phenotype in PDAC cell lines and with a dysregulation of migration, chemotaxis and cell adhesion pathways in PDAC patients, we further explored whether MRP4 modulation affects cell migration and metastatic dissemination. Our data show that suppressing MRP4 in PANC1 cells decreases cell migration in culture, which is a key step in tumor invasion and eventual formation of metastatic foci. Moreover, the transcriptomic analysis of PANC1 clones revealed that MRP4 silencing alters gene expression, mainly dysregulating pathways related to cell-to-cell interactions and focal adhesion, possibly compromising the invasive ability of PANC1 cells. MRP4sh2 cells show a lower expression of markers related to degradation and invasion of the extracellular matrix (ESRP2, PCOLCE2, LAMC3, MARCKS2, among others) and cell proliferation/survival (EGFL7, SESN2, CABLES1, MDK, among others), with a concomitant upregulation of genes associated with good prognosis in PDAC, such as BMF^33^. Furthermore, quantification of specific EMT markers, vimentin and E-cadherin, revealed MRP4 overexpression causes a switch in the expression of these two key genes, indicating a transition towards a mesenchymal phenotype in phenotypically epithelial cells, such as BxPC-3. This may translate in an augmented aggressiveness and invasive capacity. However, this does not mean that MRP4 silencing causes the reverse process, inducing mesenchymal cells, such as PANC1, to an epithelial phenotype. We speculate this might partially explain why neither E-cadherin, vimentin or GATA6 expression were significantly altered in MRP4sh cells compared to scramble cells.

To study whether MRP4 affects tumor spreading, we inoculated GFP-BxPC-3 clones into the tail vein of NSG mice and evaluated the establishment of metastatic foci in various organs. Since MRP4-overexpressing BxPC-3 cells give rise to fast growing tumors, the animals must be euthanized before spontaneous metastases are detected. Although our experimental metastatic approach is a valuable model to study certain steps of the metastatic process, we are fully aware it bypasses invasion from the primary tumor and generates colonization rather than metastasis. However, this methodology, or directly injecting PDAC cells into the spleen of mice, is commonly used to evaluate pancreatic cancer dissemination^34,35^. Together with the knowledge that CTC cells from PDAC patients express higher levels of MRP4 compared to primary tumor cells, and that silencing MRP4 alters the expression of several CTC biomarkers, such as IL11, EBI3, VEGFA, and ADGRG1^36^, our findings suggest that an upregulation of the transporter could confer an adaptive advantage associated with disease progression in PDAC patients.

Regarding the tumor colonization sites attained in our experimental model, we identified hepatic, pulmonary and renal metastatic foci, paralleling what happens in the clinic. Cancer cells originating in the pancreas preferentially metastasize to liver, lungs and peritoneum^37^, supporting the notion that pancreatic tumors are highly specific regarding the metastatic sites^38,39^. Also, as seen in our model, multiple foci sites are characteristic of hepatic metastases, and patients with liver disease often show oligometastases when diagnosed^40^. Both observations imply that our experimental approach resembles clinical findings and that it is a valuable tool to study PDAC metastatic dissemination.

Successful adhesion of tumor cells to the liver is not just a mechanical process but depends on specific interactions between the hepatic microvasculature and the cancer cells^41^. These interactions contribute to the metastasis efficacy and represent useful therapeutic targets for controlling tumor dissemination^42^. Our data show that high levels of MRP4 confer a more aggressive and invasive phenotype to PDAC cells, although its actual role upon cell migration is still controversial, as it depends on the cell system studied^43–45^. Also, it is still unclear whether MRP4 affects cell motility due to accumulation of endogenous bioactive MRP4 substrates in the extracellular compartment, such as cyclic nucleotides, prostaglandins, and leukotrienes^7,46–48^, or due to regulation of cell cycle and cytoskeleton protein levels, such as vimentin, cadherins, integrins or F-actin^49^. Regarding tumor dissemination, suppression of MRP4 showed a marked decrease in spontaneous lung metastases compared to control cells in a basal breast cancer xenograft model^50^. The authors propose MRP4 plays a crucial role in the microenvironment of the primary tumor, as it maintains high PGE2 levels which act in an autocrine or paracrine way and boost the metastatic potential and progression of the tumor. Regarding cyclic nucleotides, cAMP regulates a variety of signaling events that compose cell migration^51,52^ and affect it in a biphasic manner^53^. We recently demonstrated that modulation of MRP4 directly affects cAMP balance, determining the activation of EPAC/Rap1 signaling pathway, and impacting on PANC1 cell proliferation^9^. Accordingly, EPAC1 is often overexpressed in pancreatic cell lines and tumor samples^54^ and our in silico analysis showed that Rap1 pathway is dysregulated in MRP4 HE pancreatic carcinomas. Moreover, our RNAseq study revealed that numerous genes related to Arf6, Plexin-D1, Wnt, GPCR and PI3K/AKT cascades are differentially modulated upon MRP4 suppression and may account for MRP4sh loss in malignity. The fact that several dysregulated signaling pathways are shared in both bioinformatic analysis emphasizes our proposal that MRP4 levels are indeed associated with PDAC progression. Further studies should be performed to confirm the activation status of the mentioned pathways in MRP4sh2 and scramble cells and unravel the underlying mechanism by which MRP4 influences migration/invasive capacity in PDAC cells.

Also, as a multidrug transporter, MRP4 overexpression in tumor cells may confer resistance to anticancer agents. Notably, chemotherapeutic drugs which are commonly used in PDAC, such as gemcitabine and paclitaxel, are not MRP4 substrates^55^. However, some reports describe that MRP4 participates in the generation of chemoresistant phenotypes in cancer^56,57^. Studies involving the evaluation of the sensitivity to different anticancer agents and MRP4 expression levels will shed some light upon MRP4 role in PDAC chemoresistance and could possibly identify promising therapeutic combinations for cancer treatment.

Overall, our findings indicate that MRP4 upregulation could represent an adaptive advantage associated with poor prognosis, evidenced by the co-expression of mesenchymal markers, higher cell proliferation, tumorigenicity and invasiveness in PDAC models. Thus, we provide theoretical and experimental support for targeted treatment of pancreatic cancer by making an important contribution to the understanding of pancreatic tumor cell biology. Further studies on the structural and functional characterization of MRP4 may facilitate the design of alternative therapeutic strategies for this devastating disease.

## Materials and methods

### Cell culture

PANC1 and BxPC-3 human pancreatic cancer cell lines were obtained from the American Type Culture Collection (ATCC, USA) and grown in Dulbecco’s Modified Eagle’s Medium (DMEM) or RPMI-1640 (RPMI) medium (Sigma-Aldrich), respectively. PANC1-scramble, PANC1-MRP4sh1, PANC1-MRP4sh2, BxPC-3-mock, and BxPC-3-MRP4 cells were established and characterized in our laboratory^9^. Medium was always supplemented with 10% fetal bovine serum (FBS; Natocor, Argentina) and 50 µg/mL gentamicin (Sigma-Aldrich). All cell cultures were maintained at 37 °C in a humidified atmosphere with 5% CO_2_. PANC1 clones were maintained with puromycin from *Streptomyces alboniger* (Sigma-Aldrich; 1 µg/mL) and BxPC-3 clones were maintained with hygromycin B (SelleckChem; 200 µg/mL). MRP4 modulation does not alter cell viability in neither of the studied PDAC cell lines and MRP4sh xenografts show the same apoptotic index as scramble tumors (data not shown).

### Mice

Two-month-old male nude (Fundación Facultad de Ciencias Veterinarias, UNLP, Argentina) and NOD/LtSz-scid/IL-2Rgamma null mice (NSG; Jackson Labs, Bar Harbor, Maine, USA) were used (27 g ± 2 g). Mice were bred and maintained under specific pathogen-free conditions in filter-top boxes with a 12 h light/12 h dark cycles at the IBYME animal facility. All studies comply with the ARRIVE guidelines, were carried out in accordance with the UK Animals (Scientific Procedures) Act, 1986 and were performed according to protocols approved by the IBYME-CICUAL committee (014/2017).

### In silico analysis of MRP4/ABCC4 expression

Differential expression analysis were performed using the Gene Expression Profiling Interactive Analysis (GEPIA) server^58^ (https://gepia.cancer-pku.cn/index.html), which is based on the Cancer Genome Atlas (TCGA) and the Genotype-Tissue Expression (GTEx) project and five online datasets (GSE15471, GSE62452, GSE717296, GSE64557, and GSE18670) obtained from the Gene Expression Omnibus (GEO) database (https://www.ncbi.nlm.nih.gov/geo). GEPIA dataset contains 171 normal and 179 tumor samples, GSE15471 contains 39 tumor and paired normal samples, GSE62452 contains 61 normal and 69 tumor samples, GSE71729 contains 46 normal, 145 tumor samples and 17 pancreatic cancer cell lines, GSE64557 contains 9 PDAC cell lines, and GSE18670 contains 6 tumor and paired blood samples. As previously reported^9^, a group of 178 patients with pancreatic cancer and follow-up data (https://xena.ucsc.edu) was classified into low or high MRP4 mRNA expression levels according to the StepMiner one-step algorithm^59^. The analysis of differentially expressed genes among tumors with high MRP4 expression (n = 48; HE) and low expression (n = 24; LE) was performed using the SAM test, with the Multi Experiment Viewer software (MeV v4.9). Functional enrichment analysis of dysregulated genes was performed with the ClueGO^60^ and CluePediaCytoscape^61^ plugin based on Wikipathway, Reactome, KEGG and Gene Ontology (Go) databases.

### Stable transfection of GFP-BxPC-3 expressing cells

The p6NST50 lentiviral particles were kindly provided by Dr. Caroline Lamb from Instituto de Biología y Medicina Experimental (IBYME) and infection of BxPC-3-mock and BxPC-3-MRP4 cells was achieved as previously described^62^. Transfected clones expressing GFP were identified by fluorescence microscopy, selected with zeocin (InvivoGen; 500 μg/mL) and MRP4 overexpression was controlled by western blot analysis. For in vivo inoculation, cells were harvested using 0.25% trypsin and 0.1% EDTA (MicroVet SRL) and washed by centrifugation with serum-free RPMI medium. High GFP-expressing clones were then sorted using a FACS Aria II (BD Biosciences) and injected by tail vein in NSG mice.

### RNA isolation and quantitative real-time PCR (qPCR)

Briefly, total RNA was isolated from plate cultured cells using Quick-Zol (Kalium Technologies) and cDNA libraries were constructed from 1 µg of total RNA (M-MLV 200 U/µL; Promega) following manufacturer’s instructions. For qPCR, HOT FIREPol’ EvaGreen qPCR Mix Plus (Solis Biodyne, Tartu, Estonia) was used in a 15 µL final volume reaction. The specific PCR primer pairs used to amplify mRNA for CDH1 and vimentin genes are listed: CDH1: Fw 5′-AAGTGCTGCAGCCAAAGACAGA-3′ and Rv: 5′-AGGTAGACCCACCTCAATCATCCTC-3′; vimentin Fw: 5′-CCAGGCAAAGCAGGAGTC-3′ and Rv: 5′-CGAAGGTGACGAGCCATT-3′. Reactions were performed as follows: 95 °C for 15 min, and 40 cycles of 95 °C for 15 s, 60 °C for 20 s and 72 °C for 30 s. Values plotted are the changes relative to control (ΔΔCt), which determines the fold increase in mRNA levels relative to the internal control β-actin (Fw: 5′-GGACTTCGAGCAAGAGATGG-3′ and Rv: 5´-AGCACTGTGTTGGCGTACAG-3′). Biological triplicates were performed.

### RNAsequencing (RNAseq) and RNAseq analysis of PANC1, PANC1-scramble and PANC1-MRP4sh clones

RNA concentration and integrity were measured on an Agilent 2100 Bioanalyzer (Agilent Technologies). mRNA from PANC1, PANC1-scramble and PANC1-MRP4sh2 cells was processed for directional RNAseq library construction using the TruSeq RNA Sample Preparation Kit v2 according to the manufacturer’s protocol. We performed 101-nt paired-end sequencing using an Illumina HiSeq2500 platform at Macrogen Core Facility and obtained about 30 million tags per sample. QC and alignment of the short-read sequences against the human reference genome (hg19) was performed using the ShortRead^63^ and Rsubread^64^ R/Bioconductor (https://bioconductor.org/) packages, respectively. Subsequently, the number of reads mapped to each gene on the basis of the UCSC.hg19.KnownGene database were counted, reported, and annotated using the GenomicFeatures, GenomicAlignments, and org.Hs.eg.db packages. Raw datasets have been submitted to NCBI GEO database (Supplementary Table S2). To identify differentially expressed genes between PANC1 clones, we used the edgeR-test on the basis of the normalized number of reads mapped to each gene^65^. Functional enrichment analyses were performed using the Enrichr resource^66^ (https://amp.pharm.mssm.edu/Enrichr) and InnateDB^67^ (https://www.innatedb.com) based on the list of dysregulated transcripts between scramble and MRP4sh cells (FDR < 0.05; Fold changes > 2). Data integration and visualization of differentially expressed transcripts were done with R/Bioconductor and the MultiExperiment Viewer software^68^*.* Supplementary Table S3 condenses the top 10 down- and upregulated transcripts in MRP4sh cells compared to scramble cells. Differential expression was established according to the Log Fold Changes. The associated function for each transcript was obtained from Genecards (https://www.genecards.org) or Pub Med (https://pubmed.ncbi.nlm.nih.gov/22155432).

### Wound-healing assay

PANC1-scramble, PANC1-MRP4sh1 and PANC1-MRP4sh2 cells at exponential growth phase were seeded in 24-well plates at 1 × 10^5^ cells/well. When full confluence was reached, cells were incubated overnight in serum-free DMEM medium for cell cycle synchronization. Wounds were made by scraping the monolayer with a 200 µL pipette tip and washed twice with PBS. Cells were then incubated for 48 h in 1% FBS-supplemented medium. Images of the scratches were acquired using an Eclipse E200 (Nikon) microscope at the beginning (A_0_) and end (A_f_) of the experiment and wound healing areas were quantified using the ImageJ software (National Institutes of Health, USA). Percentage decrease of initial scratch area was calculated as 100 × (A_0_ − A_f_)/A_0_.

### In vivo assays

PANC1 and BxPC-3 clones at exponential growth phase were harvested using 0.25% trypsin and 0.1% EDTA (MicroVet SRL), and washed with PBS prior to injection. A total of 33 nude mice were randomly divided into three groups (n = 11), each inoculated subcutaneously (7 × 10^6^ cells in 100 μL DMEM) with either PANC1-scramble, PANC1-MRP4sh1 or PANC1-MRP4sh2. A total of 10 NSG mice (Jackson Labs, Bar Harbor and bread in IBYME facilities) were randomly divided into two groups (n = 5), each inoculated subcutaneously (2 × 10^6^ cells in 100 μL RPMI) with either BxPC-3-mock or BxPC-3-MRP4 cells. Tumor growth was measured three times a week using a Vernier caliper, calculating tumor volumes according to: 4/3 × π × minor radius^2^ × major radius. At the end of the experiments, mice were humanely euthanized by dislocation and tumors excised, weighed, fixed in 10% buffered formalin and embedded in paraffin for histological evaluation and immunohistochemical studies. For the experimental metastasis studies, single cell suspensions of GFP-BxPC-3-mock or GFP-BxPC-3-MRP4 cells (5 × 10^5^ in 100 μL RPMI) were injected by tail vein using a 29 gauge needle. All 21 NSG mice were monitored periodically and, after four weeks, the animals were humanely euthanized by cervical dislocation and processed as mentioned.

### Murine tissue collection and analysis of metastatic burden

All major organs were observed directly, images were captured, and location and number of metastases were recorded for each mouse. At least one metastatic lesion needed to be present for an organ to be considered positive for metastases. Lungs, liver, and kidneys were removed and visually inspected. All organs were fixed in 10% buffered formalin and embedded in paraffin before sectioning and stained with hematoxylin and eosin (H&E) according to standard protocols. Histological examination of the tumor and organ sections was performed using an Eclipse E800 light microscope (Nikon) and images were acquired with the ACT-2U software (v1.7).

### Fluorescence imaging of frozen sections

Resected lungs and kidneys were washed with PBS, fixed in cold 4% PFA for 48 h and then incubated in cold 20% sucrose for another 48 h. The organs were then embedded in OCT compound (Tissue-Tek) and immediately frozen to − 80 °C. Tissue serial sections (8 μm) were made with a Cryotome Ecryostat (Thermo) and counterstained with propidium iodide (PI, Sigma-Aldrich; 20 μg/mL). Images were acquired with an Olympus IX83 microscope and the Cell Sens Dimensions (Olympus) software.

### Tissue dissociation and flow cytometry (FAC) sample preparation

Lung and liver pieces were minced with a scalpel and digested in 4 mL of enzymatic solution (trypsin: 2.5 mg/mL and collagenase type II [Gibco BRL]: 850 U/mL in PBS) at 37 °C for 1 h with gentle agitation. The homogenates were filtered with a 70 μm nylon cell strainer to remove debris. The single cell suspensions were centrifuged for 5 min and fixed in 4% PFA overnight, washed twice in FAC buffer (PBS with 1% BSA and 0.5% sodium azide) and resuspended in FAC buffer with PI 20 μg/mL to stain cells. FAC analysis was conducted using a FACS Canto II (BD Biosciences). Cells were excited by a blue 488 nm laser (Coherent Sapphire Solid State), and GFP cells were acquired in FL1 channel (E detector) loaded with a 530/30 nm bandpass filter previously filtered with a 502 nm longpass dichroic mirror. List mode data were obtained from at least 30,000 cells and the % of GFP + cells in each homogenate was estimated. Different gates for exclusion of cell debris, duplets and quantification of GFP expression were made using the FlowJo 10 software (Tree Star, Ashland, OR, USA).

### Immunohistochemistry

Immunohistochemistry (IHC) was performed as previously described^62^, primary and secondary antibodies were used at 1/100 and 1/400 dilutions, respectively. Anti-Ki67 (ab-15580) and anti-EGFR (ab-52894) were purchased from Abcam, and anti-MRP4 (D1Z3W) and anti-GFP (D5.1 XR-P) were purchased from Cell Signaling Technology. IHC staining was reviewed in detail and a blind semi-quantitative scoring was performed by an experienced pathologist (MM). The intensity of the staining was graded as negative (0), weak (1), moderate (2), and strong (3). The staining score (scale: 0–300) results from the product between positivity (0–100%) and intensity.

### Statistical analysis

All statistical analyses were performed using Prism 6.0 software (GraphPad, San Diego, CA, USA) and, unless otherwise indicated, were carried out by either implementing Student’s *t*-test, one-way analysis of variance (ANOVA) followed by Tukey’s *t*-test or two-way ANOVA followed by Bonferroni’s multiple comparison test. Details of in silico data analyses, sample size and quantifications performed in this study are described in the corresponding “Materials and methods” section and figure legends. Experimental results are expressed as mean ± SEM and values of *p* < 0.05 were considered statistically significant.

## Supplementary information

Supplementary Information.
