# Supplementary material for: Multidrug transporter MRP4/ABCC4 as a key determinant of pancreatic cancer aggressiveness
Source: Sci Rep. 2020 Aug 26;10:14217. doi: 10.1038/s41598-020-71181-w (PMC7450045; doi:10.1038/s41598-020-71181-w)
Supplement: Supplementary file 1 — Supplementary Information. [file 41598_2020_71181_MOESM1_ESM.pdf]

Supplementary Information

Multidrug transporter MRP4/ABCC4 as a key determinant of pancreatic cancer aggressiveness

Authors: Sahores A [1], Carozzo A [1], May M [1], Gómez N [1], Di Siervi N [1], De Sousa Serro M [1], Yaneff A [1], Rodríguez-González A [2], Abba M [3], Shayo C [2], and Davio C *[1]

[1] Instituto de Investigaciones Farmacológicas (ININFA-UBA-CONICET), Facultad de Farmacia y Bioquímica, Universidad de Buenos Aires, Buenos Aires, Argentina.

[2] Instituto de Biología y Medicina (IBYME-CONICET), Buenos Aires, Argentina.

[3] Centro de Investigaciones Inmunológicas Básicas y Aplicadas, Facultad de Ciencias Médicas, Universidad Nacional de La Plata. Buenos Aires, Argentina.

* Correspondence: Dr. Carlos Davio, Instituto de Investigaciones Farmacológicas (ININFA-UBA-CONICET), Facultad de Farmacia y Bioquímica, Universidad de Buenos Aires, Junín 956, C1113AAD, Argentina. Phone: +54 11 5287 4526. E-mail: cardavio@ffyb.uba.ar

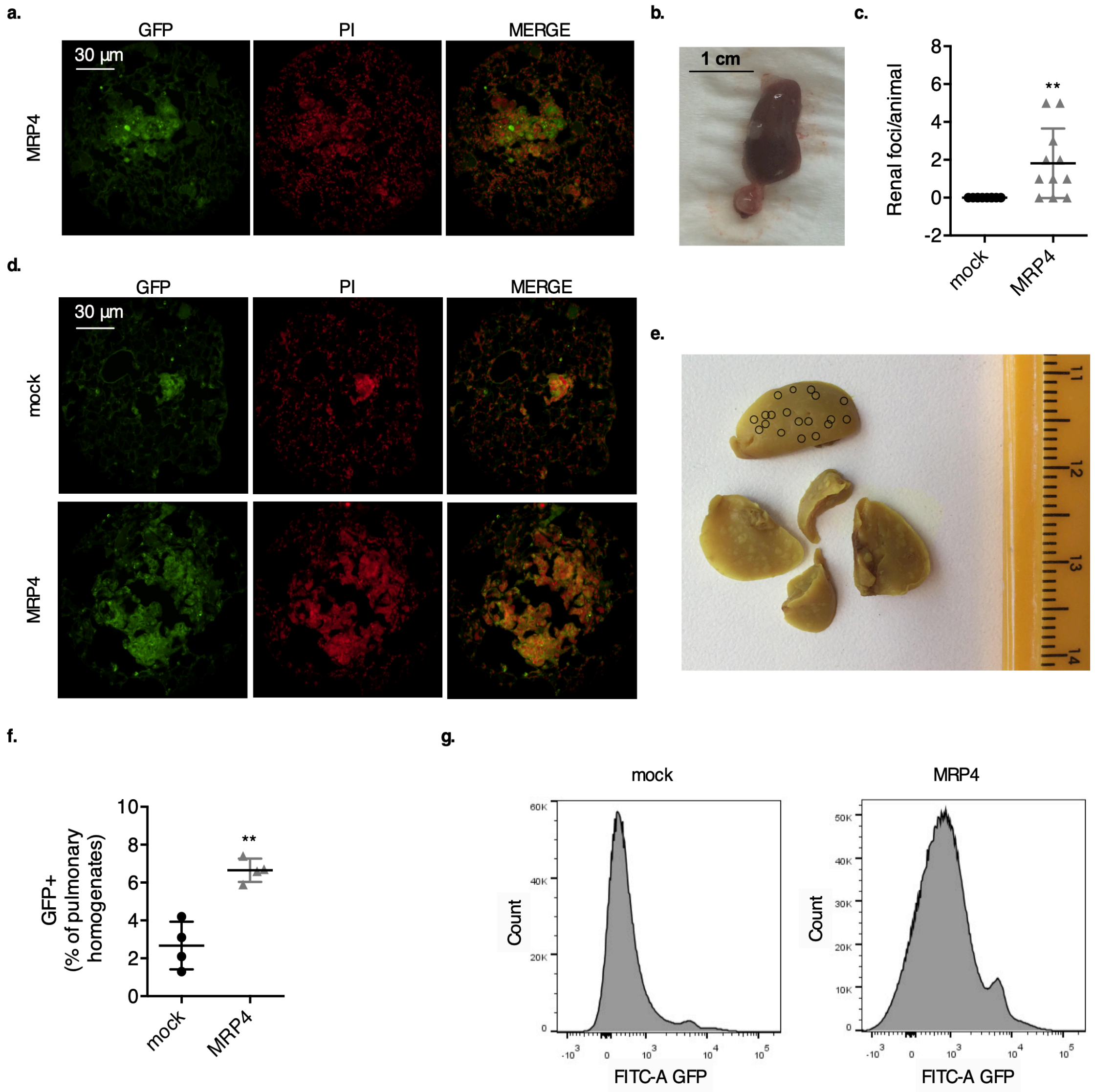

Figure S1. Effect of MRP4 overexpression upon BxPC-3 experimental metastasis. (a) Endogenous GFP fluorescence (in green) from renal metastases visualized in kidney cryostat sections of mice inoculated with MRP4-overexpressing cells. Tissues were counterstained with propidium iodide (red; PI). (b) Representative kidney with one visible metastasis. (c) Number of renal metastatic foci per animal in each experimental group. (d) Endogenous GFP fluorescence from pulmonary micro-metastases visualized in lung cryostat sections of mice from both experimental groups. (e) Representative lungs stained with Bouin solution to visualize micrometastatic foci with the naked eye. The black circles show multiple pulmonary micro-metastases. (f) Percentage of GFP+ cells in lung homogenates from both experimental groups, determined by FAC. (g) Representative comparison of GFP expression in lung homogenates, determined by FAC. Statistics: Student's t-test. (**) $p<0.01$.

Supplementary Table 1. Differentially expressed genes between MRP4 high expression carcinomas compared with low MRP4 counterparts.

Gene	Expected score (dExp)	Observed score(d)	Numerator (r)	Denominator (s+s0)	Fold change	Modulation
SCARA5	0.540	6.816	4.101	0.602	26.74	Up in ABCC4 high
IGF1	0.067	8.111	4.029	0.497	22.61	Up in ABCC4 high
CHRDL1	0.523	7.478	3.969	0.531	17.66	Up in ABCC4 high
MYOCD	0.227	6.532	2.829	0.433	14.43	Up in ABCC4 high
SYNPO2	-0.301	6.979	2.593	0.371	13.75	Up in ABCC4 high
OGN	-0.373	7.422	4.017	0.541	12.67	Up in ABCC4 high
SCN7A	-0.139	7.250	3.934	0.543	12.10	Up in ABCC4 high
PDZRN4	0.151	7.612	3.016	0.396	11.94	Up in ABCC4 high
PREX2	2.221	9.528	3.340	0.351	10.75	Up in ABCC4 high
IL7R	0.172	7.037	3.610	0.513	10.62	Up in ABCC4 high
DCC	0.517	6.520	2.023	0.310	10.09	Up in ABCC4 high
ITGA8	-2.582	9.326	3.464	0.371	9.91	Up in ABCC4 high
NRK	0.035	7.563	3.259	0.431	9.55	Up in ABCC4 high
C7	0.111	6.678	2.982	0.447	9.39	Up in ABCC4 high
ANGPTL1	0.331	7.567	3.115	0.412	9.32	Up in ABCC4 high
RSPO1	-0.041	7.270	2.863	0.394	9.25	Up in ABCC4 high
CD84	-0.026	8.249	3.238	0.393	9.06	Up in ABCC4 high
PLN	1.170	6.666	2.783	0.417	8.85	Up in ABCC4 high
ADAMTSL3	-0.398	9.904	3.273	0.330	8.70	Up in ABCC4 high
AR	-0.125	9.092	3.195	0.351	8.69	Up in ABCC4 high
RSPO3	-0.042	6.844	2.965	0.433	8.59	Up in ABCC4 high
FOXP2	-1.170	9.981	3.260	0.327	8.26	Up in ABCC4 high
CR1	-0.726	7.447	3.319	0.446	8.25	Up in ABCC4 high
ZBTB16	-0.580	7.580	3.214	0.424	8.21	Up in ABCC4 high
GLP2R	-0.130	7.036	3.078	0.437	8.17	Up in ABCC4 high
MYLK	1.860	7.676	2.521	0.328	8.16	Up in ABCC4 high
SNORD116-4	2.106	7.802	3.468	0.445	8.14	Up in ABCC4 high
RELN	0.057	6.712	3.648	0.544	8.07	Up in ABCC4 high
TNFSF8	0.991	8.800	2.920	0.332	8.06	Up in ABCC4 high
DDR2	0.104	8.844	3.255	0.368	8.03	Up in ABCC4 high
HGF	0.537	7.738	2.957	0.382	8.02	Up in ABCC4 high
P2RY14	0.347	8.804	2.796	0.318	8.00	Up in ABCC4 high
CD163	0.679	7.689	3.025	0.393	7.93	Up in ABCC4 high
SLIT2	0.952	8.755	3.556	0.406	7.87	Up in ABCC4 high
COL14A1	-1.507	8.738	2.915	0.334	7.85	Up in ABCC4 high
RGS18	1.878	8.706	2.817	0.324	7.83	Up in ABCC4 high
TLR8	-0.899	7.888	2.991	0.379	7.72	Up in ABCC4 high
PTPRC	-0.864	8.193	3.150	0.384	7.71	Up in ABCC4 high
LRRK2	0.136	9.463	3.141	0.332	7.71	Up in ABCC4 high
LAX1	-0.173	7.063	2.754	0.390	7.70	Up in ABCC4 high
KCNA3	-0.960	8.993	2.774	0.308	7.69	Up in ABCC4 high
PDE3A	-0.339	10.096	3.429	0.340	7.66	Up in ABCC4 high
SVEP1	0.406	7.115	2.821	0.396	7.54	Up in ABCC4 high
IKZF3	2.357	7.068	2.839	0.402	7.49	Up in ABCC4 high
THEMIS	0.729	6.721	2.704	0.402	7.47	Up in ABCC4 high
ASAM	-0.661	7.267	3.291	0.453	7.41	Up in ABCC4 high
OMD	-0.765	6.678	3.456	0.518	7.40	Up in ABCC4 high
NHSL2	-0.142	11.475	2.926	0.255	7.36	Up in ABCC4 high
F13A1	-0.202	7.779	2.986	0.384	7.29	Up in ABCC4 high
AOX1	-0.711	8.843	3.349	0.379	7.24	Up in ABCC4 high

Supplementary Table 1. Differentially expressed genes between MRP4 high expression carcinomas compared with low MRP4 counterparts.

Gene	Expected score (dExp)	Observed score(d)	Numerator (r)	Denominator (s+s0)	Fold change	Modulation
COL29A1	-0.137	6.635	2.589	0.390	7.20	Up in ABCC4 high
SLC18A2	-1.445	7.679	2.801	0.365	7.17	Up in ABCC4 high
FAM70A	-1.453	8.760	2.996	0.342	7.08	Up in ABCC4 high
KLHL4	0.124	9.497	2.775	0.292	7.05	Up in ABCC4 high
FGF7	1.095	7.287	2.571	0.353	7.03	Up in ABCC4 high
CXCL12	-0.450	7.970	2.557	0.321	7.01	Up in ABCC4 high
FPR2	-1.280	6.682	2.923	0.437	6.97	Up in ABCC4 high
ZNF366	-1.194	11.168	2.882	0.258	6.97	Up in ABCC4 high
C7orf58	-0.746	8.376	2.817	0.336	6.94	Up in ABCC4 high
CD226	0.685	7.916	2.543	0.321	6.89	Up in ABCC4 high
**ABCC4**	**-3.896**	**19.260**	**2.855**	**0.148**	**6.88**	**Up in ABCC4 high**
CLSTN2	0.201	8.040	2.465	0.307	6.88	Up in ABCC4 high
ABCD2	0.027	9.136	2.592	0.284	6.88	Up in ABCC4 high
SLIT3	0.719	8.396	2.597	0.309	6.87	Up in ABCC4 high
GVIN1	0.794	7.457	2.774	0.372	6.83	Up in ABCC4 high
ADAM22	0.261	7.799	2.766	0.355	6.82	Up in ABCC4 high
GPR34	1.312	9.635	2.783	0.289	6.81	Up in ABCC4 high
LYVE1	0.088	7.452	2.627	0.353	6.79	Up in ABCC4 high
TLL1	1.015	7.670	2.765	0.361	6.78	Up in ABCC4 high
NEXN	1.317	7.910	2.590	0.327	6.73	Up in ABCC4 high
CCR2	-0.029	6.980	2.662	0.381	6.73	Up in ABCC4 high
SIGLEC6	-0.827	7.445	2.623	0.352	6.70	Up in ABCC4 high
KCND3	0.582	8.178	2.963	0.362	6.68	Up in ABCC4 high
FGF10	-0.135	6.784	2.323	0.342	6.68	Up in ABCC4 high
P2RX7	-1.905	10.212	2.806	0.275	6.54	Up in ABCC4 high
FLG	0.384	6.684	2.507	0.375	6.49	Up in ABCC4 high
PRKCB	-1.586	8.083	2.982	0.369	6.46	Up in ABCC4 high
GPR171	0.428	6.651	2.588	0.389	6.37	Up in ABCC4 high
CD36	0.777	7.461	2.661	0.357	6.36	Up in ABCC4 high
CNTN1	1.117	7.255	3.103	0.428	6.35	Up in ABCC4 high
CYP1B1	-0.387	6.890	3.058	0.444	6.29	Up in ABCC4 high
P2RY1	0.408	7.553	2.676	0.354	6.28	Up in ABCC4 high
WDFY4	1.387	7.639	3.081	0.403	6.26	Up in ABCC4 high
FMO2	1.012	6.514	2.638	0.405	6.25	Up in ABCC4 high
FAT4	-0.495	10.527	2.823	0.268	6.25	Up in ABCC4 high
CASS4	0.524	8.690	2.607	0.300	6.22	Up in ABCC4 high
DAAM2	-1.374	8.769	2.298	0.262	6.20	Up in ABCC4 high
CYSLTR1	-1.040	9.750	2.697	0.277	6.19	Up in ABCC4 high
MMP16	0.333	9.844	2.861	0.291	6.16	Up in ABCC4 high
PIK3CG	-0.646	8.180	2.862	0.350	6.14	Up in ABCC4 high
ABCA8	1.605	6.857	3.028	0.442	6.14	Up in ABCC4 high
SIGLEC8	0.023	7.044	2.765	0.393	6.11	Up in ABCC4 high
SEMA3D	-0.132	6.572	2.802	0.426	6.10	Up in ABCC4 high
PYGO1	-0.290	9.436	2.764	0.293	6.09	Up in ABCC4 high
FILIP1	1.730	9.487	2.500	0.264	6.06	Up in ABCC4 high
FAM38B	1.029	8.006	2.642	0.330	5.96	Up in ABCC4 high
PRKG1	0.325	6.926	2.554	0.369	5.93	Up in ABCC4 high
CXCR2	1.202	6.669	2.689	0.403	5.89	Up in ABCC4 high
IGFBPL1	-2.154	6.714	2.288	0.341	5.88	Up in ABCC4 high
IL6ST	-1.500	7.964	3.256	0.409	5.88	Up in ABCC4 high

Supplementary Table 1. Differentially expressed genes between MRP4 high expression carcinomas compared with low MRP4 counterparts.

Gene	Expected score (dExp)	Observed score(d)	Numerator (r)	Denominator (s+s0)	Fold change	Modulation
DCLK1	1.086	8.730	2.785	0.319	5.86	Up in ABCC4 high
THBS1	-1.213	6.505	2.749	0.423	5.83	Up in ABCC4 high
ITGA4	-2.542	10.276	2.701	0.263	5.82	Up in ABCC4 high
FPR3	-1.280	7.536	2.662	0.353	5.80	Up in ABCC4 high
RGS7BP	1.113	7.495	2.438	0.325	5.80	Up in ABCC4 high
MPEG1	1.074	8.859	2.584	0.292	5.79	Up in ABCC4 high
LILRA1	1.640	8.331	2.460	0.295	5.76	Up in ABCC4 high
COL21A1	1.377	6.602	2.332	0.353	5.73	Up in ABCC4 high
FAM13C	1.880	9.023	2.939	0.326	5.73	Up in ABCC4 high
SAMHD1	0.366	9.458	2.499	0.264	5.72	Up in ABCC4 high
PDGFRA	0.365	7.440	2.404	0.323	5.72	Up in ABCC4 high
CSRNP3	1.144	7.997	2.414	0.302	5.71	Up in ABCC4 high
CD300E	0.063	7.680	2.192	0.285	5.71	Up in ABCC4 high
LRFN5	-0.654	8.097	2.518	0.311	5.69	Up in ABCC4 high
NCAM2	0.009	7.982	2.567	0.322	5.68	Up in ABCC4 high
ANGPT1	-1.353	7.934	2.381	0.300	5.67	Up in ABCC4 high
EDIL3	-0.626	7.863	2.893	0.368	5.66	Up in ABCC4 high
RUNX1T1	-1.225	8.976	2.656	0.296	5.63	Up in ABCC4 high
TXLNB	-1.003	8.074	2.540	0.315	5.62	Up in ABCC4 high
GAPT	0.596	7.081	2.674	0.378	5.60	Up in ABCC4 high
CCBE1	0.184	7.374	2.135	0.289	5.60	Up in ABCC4 high
CELF2	1.272	10.646	2.560	0.240	5.60	Up in ABCC4 high
TFEC	-0.301	7.554	2.674	0.354	5.59	Up in ABCC4 high
SDPR	-1.095	8.897	2.247	0.253	5.57	Up in ABCC4 high
C10orf128	-0.224	9.010	2.696	0.299	5.57	Up in ABCC4 high
ABI3BP	0.591	7.029	2.692	0.383	5.56	Up in ABCC4 high
CSF2RB	-0.778	7.691	2.539	0.330	5.55	Up in ABCC4 high
MS4A7	0.276	8.060	2.495	0.309	5.55	Up in ABCC4 high
P2RY13	0.348	7.826	2.489	0.318	5.51	Up in ABCC4 high
SGCD	-0.778	8.255	2.506	0.304	5.50	Up in ABCC4 high
CCR5	-0.029	6.898	2.424	0.351	5.49	Up in ABCC4 high
DOCK2	-0.183	7.835	2.717	0.347	5.48	Up in ABCC4 high
IKZF1	2.355	7.443	2.527	0.340	5.48	Up in ABCC4 high
FHL5	1.366	7.536	2.354	0.312	5.46	Up in ABCC4 high
PCDH19	0.051	7.720	2.295	0.297	5.45	Up in ABCC4 high
TEK	0.690	10.027	2.446	0.244	5.45	Up in ABCC4 high
AOAH	-0.413	7.148	2.432	0.340	5.42	Up in ABCC4 high
CD28	-0.501	7.074	2.547	0.360	5.40	Up in ABCC4 high
ALDH1L2	-0.797	9.012	2.721	0.302	5.37	Up in ABCC4 high
HTR2A	-0.906	6.667	2.242	0.336	5.36	Up in ABCC4 high
CXorf21	1.595	7.849	2.392	0.305	5.36	Up in ABCC4 high
HRH2	-1.815	10.363	2.482	0.239	5.36	Up in ABCC4 high
HMCN1	-0.629	6.990	2.639	0.378	5.35	Up in ABCC4 high
TLR7	-0.151	7.160	2.542	0.355	5.33	Up in ABCC4 high
THSD7A	0.256	8.870	2.515	0.284	5.31	Up in ABCC4 high
MRC1	0.451	8.610	2.737	0.318	5.31	Up in ABCC4 high
GPR141	-0.890	7.269	2.096	0.288	5.30	Up in ABCC4 high
CNKSR2	1.107	7.696	2.799	0.364	5.30	Up in ABCC4 high
SULT1C4	1.367	8.726	2.414	0.277	5.30	Up in ABCC4 high
SFMBT2	-0.600	9.469	2.585	0.273	5.28	Up in ABCC4 high

Supplementary Table 1. Differentially expressed genes between MRP4 high expression carcinomas compared with low MRP4 counterparts.

Gene	Expected score (dExp)	Observed score(d)	Numerator (r)	Denominator (s+s0)	Fold change	Modulation
SUSD5	-0.315	8.659	2.426	0.280	5.25	Up in ABCC4 high
BICC1	0.421	7.243	3.250	0.449	5.19	Up in ABCC4 high
RCSD1	0.116	8.502	2.385	0.281	5.19	Up in ABCC4 high
KLHL6	0.123	6.612	2.408	0.364	5.18	Up in ABCC4 high
CNTN4	0.376	8.674	2.577	0.297	5.17	Up in ABCC4 high
ANKRD44	-0.092	9.884	2.431	0.246	5.16	Up in ABCC4 high
GIMAP6	-0.177	10.897	2.323	0.213	5.14	Up in ABCC4 high
CCR8	-0.028	6.777	2.344	0.346	5.13	Up in ABCC4 high
ADAMTSL1	-0.398	7.738	2.399	0.310	5.13	Up in ABCC4 high
CD200R1	-1.431	7.877	2.310	0.293	5.13	Up in ABCC4 high
RBMS3	0.265	7.300	2.366	0.324	5.11	Up in ABCC4 high
MS4A4A	1.153	7.167	2.495	0.348	5.08	Up in ABCC4 high
LILRB5	0.032	6.661	2.522	0.379	5.07	Up in ABCC4 high
CD38	0.775	6.773	2.869	0.424	5.07	Up in ABCC4 high
ART4	-2.268	7.180	2.422	0.337	5.06	Up in ABCC4 high
MSR1	0.930	7.097	2.515	0.354	5.04	Up in ABCC4 high
FBN1	-0.415	7.452	2.566	0.344	5.01	Up in ABCC4 high
EFEMP1	0.465	6.599	2.309	0.350	4.98	Up in ABCC4 high
EPHA3	-0.138	7.491	2.550	0.340	4.95	Up in ABCC4 high
ADAMTS3	1.220	6.833	2.290	0.335	4.95	Up in ABCC4 high
RASSF2	-0.478	8.656	2.214	0.256	4.94	Up in ABCC4 high
CALCRL	-1.665	10.112	2.435	0.241	4.91	Up in ABCC4 high
SIGLEC10	2.055	7.128	2.328	0.327	4.90	Up in ABCC4 high
PIK3R5	0.000	7.760	2.357	0.304	4.90	Up in ABCC4 high
MARCH1	0.200	8.683	2.305	0.265	4.89	Up in ABCC4 high
SIGLEC5	0.028	7.369	2.506	0.340	4.88	Up in ABCC4 high
C6orf186	-0.621	7.773	2.219	0.286	4.88	Up in ABCC4 high
ABCB1	-0.053	6.832	2.538	0.371	4.86	Up in ABCC4 high
ZNF354C	-1.001	9.406	2.687	0.286	4.85	Up in ABCC4 high
ROR1	-0.739	8.839	2.648	0.300	4.84	Up in ABCC4 high
KIRREL	1.184	7.972	3.042	0.382	4.83	Up in ABCC4 high
ATP8B4	-0.914	9.096	2.162	0.238	4.83	Up in ABCC4 high
PTGER3	1.181	6.737	2.520	0.374	4.80	Up in ABCC4 high
ITGA9	-2.578	8.333	2.495	0.299	4.79	Up in ABCC4 high
RECK	-1.229	11.965	2.326	0.194	4.79	Up in ABCC4 high
NEFH	1.184	6.566	2.144	0.327	4.77	Up in ABCC4 high
BOC	-1.201	8.970	2.311	0.258	4.76	Up in ABCC4 high
CCR1	-0.029	7.658	2.278	0.297	4.76	Up in ABCC4 high
PDE8B	0.736	6.749	2.143	0.318	4.76	Up in ABCC4 high
CYBB	-0.754	6.903	2.290	0.332	4.75	Up in ABCC4 high
GREB1	1.262	8.063	2.400	0.298	4.73	Up in ABCC4 high
P2RY12	0.348	7.036	2.471	0.351	4.73	Up in ABCC4 high
LRRC2	-0.188	7.214	2.239	0.310	4.72	Up in ABCC4 high
PLXNC1	1.560	8.786	3.179	0.362	4.71	Up in ABCC4 high
MNDA	-0.609	7.630	2.611	0.342	4.70	Up in ABCC4 high
WISP1	0.849	6.491	2.636	0.406	4.69	Up in ABCC4 high
C1QTNF7	-0.123	7.686	2.181	0.284	4.68	Up in ABCC4 high
CACNA2D1	-0.005	7.798	3.365	0.432	4.63	Up in ABCC4 high
SIRPB2	0.342	7.026	2.322	0.330	4.63	Up in ABCC4 high
ZFHX4	0.000	6.697	2.430	0.363	4.63	Up in ABCC4 high

Supplementary Table 1. Differentially expressed genes between MRP4 high expression carcinomas compared with low MRP4 counterparts.

Gene	Expected score (dExp)	Observed score(d)	Numerator (r)	Denominator (s+s0)	Fold change	Modulation
USP51	-0.486	9.883	2.486	0.252	4.62	Up in ABCC4 high
NCKAP1L	-0.421	7.303	2.398	0.328	4.62	Up in ABCC4 high
SLA	-0.534	7.080	2.266	0.320	4.59	Up in ABCC4 high
SHE	-0.760	9.898	2.204	0.223	4.57	Up in ABCC4 high
TSHZ2	-1.754	7.356	2.317	0.315	4.57	Up in ABCC4 high
EVI2A	-1.511	7.425	2.357	0.318	4.57	Up in ABCC4 high
DMGDH	0.832	8.150	2.139	0.262	4.56	Up in ABCC4 high
IL1R1	-0.466	7.762	2.269	0.292	4.54	Up in ABCC4 high
FPR1	-1.280	6.685	2.278	0.341	4.54	Up in ABCC4 high
EVI2B	-1.510	7.151	2.225	0.311	4.54	Up in ABCC4 high
ZNF518B	0.120	9.723	2.290	0.236	4.52	Up in ABCC4 high
SIRPB1	0.343	9.223	2.600	0.282	4.52	Up in ABCC4 high
ECM2	-0.517	8.116	2.262	0.279	4.51	Up in ABCC4 high
GNB4	0.393	9.793	2.245	0.229	4.49	Up in ABCC4 high
MERTK	1.273	10.799	2.225	0.206	4.49	Up in ABCC4 high
GUCY1A3	-0.144	8.515	2.415	0.284	4.49	Up in ABCC4 high
SLCO2B1	-0.031	7.778	2.105	0.271	4.49	Up in ABCC4 high
PCDH18	0.051	8.683	2.128	0.245	4.47	Up in ABCC4 high
CD209	0.791	6.607	2.121	0.321	4.44	Up in ABCC4 high
GIMAP8	-0.177	10.295	2.123	0.206	4.44	Up in ABCC4 high
ACSM5	-0.549	7.136	2.230	0.313	4.44	Up in ABCC4 high
HEG1	-0.146	11.360	2.279	0.201	4.43	Up in ABCC4 high
PAR5	-0.907	6.652	2.280	0.343	4.43	Up in ABCC4 high
MSRB3	-0.527	7.496	2.165	0.289	4.42	Up in ABCC4 high
BHLHE22	0.486	7.195	2.281	0.317	4.38	Up in ABCC4 high
PDCD1LG2	-0.137	6.626	2.175	0.328	4.38	Up in ABCC4 high
ZEB2	0.701	9.361	2.281	0.244	4.38	Up in ABCC4 high
C17orf87	1.187	7.594	2.299	0.303	4.37	Up in ABCC4 high
ZNF521	-0.293	7.912	2.169	0.274	4.37	Up in ABCC4 high
SLC9A9	-0.047	9.062	2.163	0.239	4.36	Up in ABCC4 high
NAV3	-0.171	8.136	2.343	0.288	4.35	Up in ABCC4 high
PLEK	1.452	6.974	2.222	0.319	4.35	Up in ABCC4 high
CDK15	-1.404	7.398	1.826	0.247	4.34	Up in ABCC4 high
NUDT10	-1.018	6.876	1.985	0.289	4.33	Up in ABCC4 high
GHR	-0.232	8.012	2.335	0.291	4.33	Up in ABCC4 high
CMKLR1	0.088	7.327	2.181	0.298	4.32	Up in ABCC4 high
ESR1	-0.021	7.324	2.103	0.287	4.31	Up in ABCC4 high
PGR	-0.240	6.785	2.616	0.386	4.30	Up in ABCC4 high
GPR133	0.234	7.160	2.218	0.310	4.30	Up in ABCC4 high
DOCK11	0.612	8.419	2.005	0.238	4.26	Up in ABCC4 high
UNC5C	-1.262	6.638	2.152	0.324	4.26	Up in ABCC4 high
AVPR1A	1.641	6.599	2.425	0.368	4.25	Up in ABCC4 high
RORB	-0.743	7.461	2.035	0.273	4.23	Up in ABCC4 high
C3AR1	-0.413	7.077	2.243	0.317	4.23	Up in ABCC4 high
GIMAP7	-0.176	7.680	2.018	0.263	4.21	Up in ABCC4 high
MS4A6A	0.998	7.240	2.197	0.303	4.21	Up in ABCC4 high
LPPR4	-1.599	7.170	2.133	0.298	4.20	Up in ABCC4 high
BHMT2	-1.410	7.426	2.066	0.278	4.19	Up in ABCC4 high
LEPR	0.350	7.741	2.056	0.266	4.19	Up in ABCC4 high
MPDZ	0.290	10.259	2.373	0.231	4.18	Up in ABCC4 high

Supplementary Table 1. Differentially expressed genes between MRP4 high expression carcinomas compared with low MRP4 counterparts.

Gene	Expected score (dExp)	Observed score(d)	Numerator (r)	Denominator (s+s0)	Fold change	Modulation
LOC401463	-0.872	8.374	1.926	0.230	4.16	Up in ABCC4 high
CD180	1.044	6.515	2.217	0.340	4.15	Up in ABCC4 high
ACSS3	-1.239	6.731	2.136	0.317	4.15	Up in ABCC4 high
JAM2	-0.446	9.560	2.097	0.219	4.10	Up in ABCC4 high
PCDHGA9	0.424	7.460	2.391	0.320	4.10	Up in ABCC4 high
ARHGEF6	1.220	8.180	2.017	0.247	4.09	Up in ABCC4 high
STON1	2.431	7.473	2.038	0.273	4.07	Up in ABCC4 high
S1PR1	-1.970	9.342	1.964	0.210	4.07	Up in ABCC4 high
PNOC	-2.163	6.787	2.463	0.363	4.07	Up in ABCC4 high
HLA-DOA	-0.021	7.032	2.395	0.341	4.06	Up in ABCC4 high
LCP2	-0.086	8.102	2.073	0.256	4.06	Up in ABCC4 high
KLRD1	-0.665	6.782	2.203	0.325	4.06	Up in ABCC4 high
CRISPLD1	0.058	7.225	2.089	0.289	4.05	Up in ABCC4 high
CTTNBP2	-0.458	7.059	2.247	0.318	4.02	Up in ABCC4 high
CSF1R	-0.571	7.342	2.138	0.291	4.01	Up in ABCC4 high
CD93	1.712	9.839	1.991	0.202	3.98	Up in ABCC4 high
LILRB2	0.033	7.070	2.218	0.314	3.98	Up in ABCC4 high
TCF4	0.206	10.066	1.942	0.193	3.96	Up in ABCC4 high
AKAP2	-1.478	7.409	2.621	0.354	3.94	Up in ABCC4 high
SPARCL1	-1.277	7.917	2.064	0.261	3.94	Up in ABCC4 high
PDE1A	-0.411	8.454	2.087	0.247	3.92	Up in ABCC4 high
PRTG	0.235	10.967	2.289	0.209	3.91	Up in ABCC4 high
ILDR2	0.041	7.638	2.081	0.273	3.91	Up in ABCC4 high
GLIPR1	-0.870	7.315	2.099	0.287	3.90	Up in ABCC4 high
GGTA1	0.916	7.014	2.104	0.300	3.89	Up in ABCC4 high
LIN7A	0.198	9.557	2.031	0.213	3.87	Up in ABCC4 high
LOC653653	0.758	10.298	2.174	0.211	3.87	Up in ABCC4 high
GUCY1A2	-0.144	7.213	2.099	0.291	3.84	Up in ABCC4 high
BTK	0.752	6.672	2.390	0.358	3.83	Up in ABCC4 high
TMEM150C	-0.773	8.877	2.199	0.248	3.81	Up in ABCC4 high
MEFV	-0.145	6.653	2.174	0.327	3.81	Up in ABCC4 high
GPR65	-0.335	6.734	1.970	0.292	3.81	Up in ABCC4 high
KBTBD8	-0.144	8.404	1.907	0.227	3.81	Up in ABCC4 high
SPN	-2.809	6.621	2.200	0.332	3.80	Up in ABCC4 high
RASSF8	0.983	8.148	2.257	0.277	3.80	Up in ABCC4 high
NLRC4	-0.052	7.052	1.859	0.264	3.79	Up in ABCC4 high
CD53	0.666	6.497	2.145	0.330	3.78	Up in ABCC4 high
EDNRB	-1.114	7.738	1.974	0.255	3.78	Up in ABCC4 high
DOCK10	0.611	8.092	2.403	0.297	3.78	Up in ABCC4 high
EMCN	1.160	7.226	2.028	0.281	3.78	Up in ABCC4 high
LIFR	0.685	7.466	2.082	0.279	3.77	Up in ABCC4 high
BEND6	1.587	7.017	2.018	0.288	3.77	Up in ABCC4 high
RASGRP4	0.755	8.006	1.874	0.234	3.76	Up in ABCC4 high
SIGLEC9	0.417	6.822	2.038	0.299	3.75	Up in ABCC4 high
NLRP3	0.568	6.931	1.977	0.285	3.75	Up in ABCC4 high
CALD1	0.405	7.148	1.943	0.272	3.75	Up in ABCC4 high
LILRB1	0.033	7.204	2.160	0.300	3.74	Up in ABCC4 high
RAB39	0.671	8.016	2.324	0.290	3.73	Up in ABCC4 high
PCDH17	0.216	7.661	2.166	0.283	3.72	Up in ABCC4 high
ITPR2	0.141	7.267	1.610	0.222	3.71	Up in ABCC4 high

Supplementary Table 1. Differentially expressed genes between MRP4 high expression carcinomas compared with low MRP4 counterparts.

Gene	Expected score (dExp)	Observed score(d)	Numerator (r)	Denominator (s+s0)	Fold change	Modulation
FERMT2	-0.289	8.831	2.030	0.230	3.70	Up in ABCC4 high
ZFP92	0.098	8.575	1.908	0.222	3.67	Up in ABCC4 high
LPAR4	0.894	7.676	1.818	0.237	3.67	Up in ABCC4 high
SCN4B	1.149	6.876	1.915	0.278	3.66	Up in ABCC4 high
CLEC7A	-0.975	6.703	2.244	0.335	3.66	Up in ABCC4 high
ZEB1	0.109	9.528	1.955	0.205	3.65	Up in ABCC4 high
TUB	-1.084	7.801	2.145	0.275	3.65	Up in ABCC4 high
ZNF98	-1.294	8.199	1.768	0.216	3.65	Up in ABCC4 high
TM6SF1	0.365	9.674	2.085	0.216	3.65	Up in ABCC4 high
FAM198B	-1.206	8.218	1.867	0.227	3.65	Up in ABCC4 high
RASGRF2	0.867	7.887	2.134	0.271	3.64	Up in ABCC4 high
IL6R	-1.326	7.286	2.334	0.320	3.63	Up in ABCC4 high
FLI1	0.121	8.828	2.002	0.227	3.62	Up in ABCC4 high
SHISA6	0.358	6.565	1.911	0.291	3.61	Up in ABCC4 high
RNF150	-0.132	8.054	2.544	0.316	3.60	Up in ABCC4 high
PABPC4L	-0.320	8.041	2.082	0.259	3.60	Up in ABCC4 high
GIMAP5	-0.177	7.655	1.798	0.235	3.60	Up in ABCC4 high
TRIM61	-0.767	7.062	1.911	0.271	3.59	Up in ABCC4 high
ABCA9	1.606	7.080	2.430	0.343	3.58	Up in ABCC4 high
CYYR1	0.059	9.289	1.877	0.202	3.58	Up in ABCC4 high
IL10RA	-2.171	7.881	1.935	0.245	3.56	Up in ABCC4 high
MAN1A1	1.852	8.245	2.343	0.284	3.55	Up in ABCC4 high
FKBP5	1.513	7.274	2.081	0.286	3.55	Up in ABCC4 high
ERG	-0.419	8.974	1.963	0.219	3.55	Up in ABCC4 high
CLIC2	-1.925	7.729	1.973	0.255	3.55	Up in ABCC4 high
PTPLAD2	-0.979	7.008	2.025	0.289	3.54	Up in ABCC4 high
GIMAP4	-0.177	8.211	1.844	0.225	3.53	Up in ABCC4 high
ZNF727	-1.237	7.194	2.085	0.290	3.52	Up in ABCC4 high
EVC	0.076	6.737	2.054	0.305	3.51	Up in ABCC4 high
ABCA6	1.600	6.740	2.449	0.363	3.51	Up in ABCC4 high
CYP7B1	0.086	6.677	2.392	0.358	3.50	Up in ABCC4 high
BCL2	-0.930	7.785	1.811	0.233	3.50	Up in ABCC4 high
P2RY8	-1.397	7.068	1.886	0.267	3.50	Up in ABCC4 high
PRICKLE1	0.819	7.630	2.198	0.288	3.49	Up in ABCC4 high
FAM124B	-0.698	6.960	1.741	0.250	3.48	Up in ABCC4 high
STK32B	0.622	6.889	1.958	0.284	3.48	Up in ABCC4 high
HLA-DPA1	-1.900	6.779	2.056	0.303	3.48	Up in ABCC4 high
SIGLECP3	0.654	6.706	1.962	0.293	3.48	Up in ABCC4 high
KCNT2	0.312	8.220	2.363	0.287	3.48	Up in ABCC4 high
PCDHGA12	1.055	6.607	2.103	0.318	3.46	Up in ABCC4 high
SACS	0.146	10.458	2.163	0.207	3.44	Up in ABCC4 high
PDE4B	1.995	7.870	1.812	0.230	3.43	Up in ABCC4 high
STARD8	0.170	9.568	1.855	0.194	3.43	Up in ABCC4 high
EML1	-0.045	10.003	2.009	0.201	3.42	Up in ABCC4 high
CD1D	0.898	7.077	1.802	0.255	3.42	Up in ABCC4 high
C21orf34	-0.543	6.494	1.809	0.279	3.41	Up in ABCC4 high
ITPRIPL1	1.370	6.596	1.781	0.270	3.41	Up in ABCC4 high
ITPR1	0.141	9.052	2.008	0.222	3.40	Up in ABCC4 high
PPP1R16B	0.129	7.266	1.837	0.253	3.40	Up in ABCC4 high
HIPK3	0.690	6.848	2.329	0.340	3.40	Up in ABCC4 high

Supplementary Table 1. Differentially expressed genes between MRP4 high expression carcinomas compared with low MRP4 counterparts.

Gene	Expected score (dExp)	Observed score(d)	Numerator (r)	Denominator (s+s0)	Fold change	Modulation
TTBK2	0.233	7.825	2.069	0.264	3.38	Up in ABCC4 high
FGF2	1.096	6.901	2.091	0.303	3.38	Up in ABCC4 high
FAM124A	-0.698	7.581	1.742	0.230	3.38	Up in ABCC4 high
KCNN3	-0.642	8.227	2.002	0.243	3.38	Up in ABCC4 high
GLT8D2	-0.939	7.562	1.888	0.250	3.37	Up in ABCC4 high
PLCL1	1.575	10.014	2.136	0.213	3.36	Up in ABCC4 high
ARL10	-0.613	9.042	1.810	0.200	3.36	Up in ABCC4 high
ABCC9	2.064	8.181	2.852	0.349	3.36	Up in ABCC4 high
ARHGAP20	-1.626	7.731	2.107	0.273	3.35	Up in ABCC4 high
FAM26E	0.830	6.729	1.895	0.282	3.35	Up in ABCC4 high
DLC1	0.011	9.129	1.728	0.189	3.33	Up in ABCC4 high
LOC339524	-0.363	6.846	1.925	0.281	3.32	Up in ABCC4 high
SRGN	-1.186	6.878	1.903	0.277	3.31	Up in ABCC4 high
MEF2C	-1.080	8.804	1.830	0.208	3.31	Up in ABCC4 high
USP44	-0.296	6.907	1.931	0.280	3.31	Up in ABCC4 high
FHL1	1.365	6.536	1.798	0.275	3.30	Up in ABCC4 high
RGS22	0.048	6.701	2.284	0.341	3.30	Up in ABCC4 high
AKAP12	1.180	7.212	2.153	0.298	3.29	Up in ABCC4 high
ZNF804A	0.000	6.748	1.812	0.269	3.29	Up in ABCC4 high
MRVI1	-0.079	6.548	1.781	0.272	3.27	Up in ABCC4 high
DOCK4	-0.182	10.783	1.746	0.162	3.27	Up in ABCC4 high
RHOJ	0.910	8.614	1.835	0.213	3.27	Up in ABCC4 high
MYO5A	1.683	9.260	2.036	0.220	3.26	Up in ABCC4 high
C14orf49	-0.264	6.802	1.852	0.272	3.26	Up in ABCC4 high
OLFML1	0.845	6.674	1.887	0.283	3.25	Up in ABCC4 high
GAB3	-0.769	7.564	1.877	0.248	3.25	Up in ABCC4 high
RFTN2	0.387	8.196	1.655	0.202	3.23	Up in ABCC4 high
TAOK1	-0.744	7.044	1.963	0.279	3.23	Up in ABCC4 high
APOLD1	-0.033	7.315	1.663	0.227	3.23	Up in ABCC4 high
KDR	2.513	8.677	1.884	0.217	3.23	Up in ABCC4 high
TAL1	-0.169	7.859	1.722	0.219	3.22	Up in ABCC4 high
WNT9B	-0.251	6.889	1.723	0.250	3.21	Up in ABCC4 high
NID2	-0.599	6.933	1.914	0.276	3.21	Up in ABCC4 high
TRIM63	1.530	6.626	1.786	0.270	3.20	Up in ABCC4 high
SYNE1	1.474	8.560	1.871	0.219	3.19	Up in ABCC4 high
GRAP2	0.033	6.931	1.886	0.272	3.19	Up in ABCC4 high
STAT4	-1.375	6.589	1.781	0.270	3.19	Up in ABCC4 high
LOC100190938	0.423	7.100	1.711	0.241	3.18	Up in ABCC4 high
MYO9A	1.277	7.666	1.954	0.255	3.18	Up in ABCC4 high
FGL2	0.174	7.205	2.382	0.331	3.17	Up in ABCC4 high
CD4	1.942	6.542	1.741	0.266	3.17	Up in ABCC4 high
GYPE	-0.113	7.779	1.715	0.220	3.17	Up in ABCC4 high
PREX1	1.504	8.392	1.732	0.206	3.17	Up in ABCC4 high
ADAMTS2	-0.344	6.761	2.045	0.302	3.16	Up in ABCC4 high
PALMD	-1.354	6.488	1.749	0.270	3.15	Up in ABCC4 high
COL15A1	-1.123	6.555	1.987	0.303	3.15	Up in ABCC4 high
MYCT1	-0.160	8.015	1.699	0.212	3.15	Up in ABCC4 high
PDE7B	-0.153	6.746	1.717	0.254	3.14	Up in ABCC4 high
FIGN	-0.785	7.457	1.880	0.252	3.13	Up in ABCC4 high
ARHGAP31	0.087	8.543	1.588	0.186	3.13	Up in ABCC4 high

Supplementary Table 1. Differentially expressed genes between MRP4 high expression carcinomas compared with low MRP4 counterparts.

Gene	Expected score (dExp)	Observed score(d)	Numerator (r)	Denominator (s+s0)	Fold change	Modulation
DIXDC1	0.958	9.937	1.622	0.163	3.12	Up in ABCC4 high
SHROOM4	0.707	8.966	1.674	0.187	3.12	Up in ABCC4 high
ITGA1	-2.555	7.835	1.749	0.223	3.12	Up in ABCC4 high
N4BP2	-0.828	6.662	1.924	0.289	3.11	Up in ABCC4 high
WIPF1	0.088	6.777	1.608	0.237	3.11	Up in ABCC4 high
PKD2	1.137	9.135	1.846	0.202	3.11	Up in ABCC4 high
DZIP1	1.017	8.284	2.092	0.253	3.11	Up in ABCC4 high
FBXL7	-0.910	7.705	1.765	0.229	3.10	Up in ABCC4 high
CD302	0.146	8.544	1.553	0.182	3.10	Up in ABCC4 high
SLC30A4	-0.046	8.529	1.829	0.214	3.07	Up in ABCC4 high
LDLRAD3	-0.184	7.374	1.891	0.256	3.07	Up in ABCC4 high
UTRN	-0.707	10.163	1.692	0.166	3.07	Up in ABCC4 high
FAM168A	0.072	7.615	1.806	0.237	3.07	Up in ABCC4 high
GAS7	0.364	7.691	1.969	0.256	3.07	Up in ABCC4 high
ATP8B2	1.360	9.351	1.875	0.200	3.07	Up in ABCC4 high
RERG	-0.375	6.574	1.946	0.296	3.07	Up in ABCC4 high
FSTL1	-0.154	6.620	1.843	0.278	3.06	Up in ABCC4 high
ODZ3	-0.876	6.688	2.323	0.347	3.05	Up in ABCC4 high
ZNF423	0.741	6.997	1.747	0.250	3.05	Up in ABCC4 high
TMTC1	-0.260	7.483	2.131	0.285	3.04	Up in ABCC4 high
STOX2	2.563	6.515	1.924	0.295	3.03	Up in ABCC4 high
LRRC25	-0.616	6.646	1.772	0.267	3.02	Up in ABCC4 high
GJA5	-1.087	6.976	1.624	0.233	3.02	Up in ABCC4 high
MFAP3L	-1.196	7.102	1.761	0.248	3.02	Up in ABCC4 high
CPEB1	-2.200	7.380	2.318	0.314	3.01	Up in ABCC4 high
PRKAR2B	-0.357	6.613	1.952	0.295	3.01	Up in ABCC4 high
BACH2	-0.634	7.116	1.922	0.270	3.01	Up in ABCC4 high
TNFRSF19	-0.732	6.779	2.071	0.305	3.01	Up in ABCC4 high
SYT15	-0.601	6.536	1.443	0.221	3.00	Up in ABCC4 high
KLF8	1.676	6.904	1.675	0.243	3.00	Up in ABCC4 high
PRKD1	-0.461	7.137	1.926	0.270	3.00	Up in ABCC4 high
NEGR1	-0.269	6.987	2.037	0.292	3.00	Up in ABCC4 high
ABCA1	1.602	8.434	1.752	0.208	3.00	Up in ABCC4 high
KCTD12	-0.152	8.578	1.718	0.200	2.99	Up in ABCC4 high
AMOTL1	0.987	8.751	1.852	0.212	2.99	Up in ABCC4 high
C6orf204	0.558	7.119	1.644	0.231	2.99	Up in ABCC4 high
GPC6	-0.654	6.548	1.803	0.275	2.99	Up in ABCC4 high
GJD3	-0.044	7.394	1.624	0.220	2.98	Up in ABCC4 high
PPAP2B	1.145	8.803	1.615	0.183	2.97	Up in ABCC4 high
MAP7D3	-1.175	7.797	1.833	0.235	2.97	Up in ABCC4 high
LOC100272216	0.056	6.883	1.863	0.271	2.96	Up in ABCC4 high
GPR63	-0.335	8.042	1.576	0.196	2.96	Up in ABCC4 high
RAB23	0.222	8.070	1.578	0.195	2.95	Up in ABCC4 high
NAALAD2	-0.744	6.532	1.523	0.233	2.95	Up in ABCC4 high
ZBED3	-0.025	8.472	1.679	0.198	2.95	Up in ABCC4 high
PALLD	0.263	7.091	1.671	0.236	2.95	Up in ABCC4 high
ZNF660	0.638	8.924	1.812	0.203	2.95	Up in ABCC4 high
SH3BP5	-0.592	7.079	1.530	0.216	2.95	Up in ABCC4 high
CHL1	-0.848	6.809	3.044	0.447	2.94	Up in ABCC4 high
CYP26B1	0.212	6.666	1.838	0.276	2.94	Up in ABCC4 high

Supplementary Table 1. Differentially expressed genes between MRP4 high expression carcinomas compared with low MRP4 counterparts.

Gene	Expected score (dExp)	Observed score(d)	Numerator (r)	Denominator (s+s0)	Fold change	Modulation
RNF180	-1.906	7.107	1.868	0.263	2.94	Up in ABCC4 high
PPM1K	0.100	7.869	1.557	0.198	2.93	Up in ABCC4 high
LHFP	-1.180	6.772	1.588	0.235	2.93	Up in ABCC4 high
GJC1	-1.542	8.541	1.610	0.189	2.92	Up in ABCC4 high
RFPL1S	-0.625	6.630	1.655	0.250	2.92	Up in ABCC4 high
SNED1	0.255	8.504	1.583	0.186	2.91	Up in ABCC4 high
LMBRD2	-1.489	7.942	1.599	0.201	2.91	Up in ABCC4 high
MCTP1	-0.573	7.517	2.238	0.298	2.89	Up in ABCC4 high
ZNF677	0.326	6.851	1.779	0.260	2.89	Up in ABCC4 high
REV3L	-1.052	9.994	1.719	0.172	2.89	Up in ABCC4 high
PTPRG	-0.865	8.694	1.687	0.194	2.89	Up in ABCC4 high
ELTD1	-1.514	9.176	1.726	0.188	2.88	Up in ABCC4 high
RAD54L2	0.693	6.846	1.812	0.265	2.87	Up in ABCC4 high
CDH5	1.224	8.699	1.650	0.190	2.86	Up in ABCC4 high
CLIC4	-1.927	6.910	1.535	0.222	2.85	Up in ABCC4 high
CCDC36	-0.954	6.676	1.522	0.228	2.85	Up in ABCC4 high
TMEM26	-0.662	6.605	1.600	0.242	2.85	Up in ABCC4 high
MYH10	-0.254	7.898	1.625	0.206	2.85	Up in ABCC4 high
LRP12	-0.656	7.987	1.821	0.228	2.85	Up in ABCC4 high
SIRPA	0.028	9.135	1.824	0.200	2.84	Up in ABCC4 high
GPR124	-1.404	8.653	1.727	0.200	2.82	Up in ABCC4 high
RGL1	-0.374	8.713	1.636	0.188	2.82	Up in ABCC4 high
LCA5	-0.751	9.842	1.653	0.168	2.82	Up in ABCC4 high
NUDT11	-0.849	6.982	1.677	0.240	2.82	Up in ABCC4 high
LRCH2	0.105	8.218	2.099	0.255	2.81	Up in ABCC4 high
TTC28	-1.331	10.350	1.532	0.148	2.81	Up in ABCC4 high
VASH2	1.374	6.519	1.571	0.241	2.80	Up in ABCC4 high
GUCY1B3	1.330	7.422	1.872	0.252	2.80	Up in ABCC4 high
NID1	-0.597	6.885	1.665	0.242	2.80	Up in ABCC4 high
AGTR1	1.931	6.691	2.012	0.301	2.80	Up in ABCC4 high
ZSCAN23	1.052	6.506	2.054	0.316	2.79	Up in ABCC4 high
EFHA2	-2.061	6.587	1.957	0.297	2.79	Up in ABCC4 high
PXK	0.312	9.668	1.549	0.160	2.79	Up in ABCC4 high
QKI	-0.815	7.767	1.583	0.204	2.79	Up in ABCC4 high
PLAGL1	0.959	8.223	1.914	0.233	2.78	Up in ABCC4 high
GATSL1	-0.489	6.939	2.037	0.293	2.78	Up in ABCC4 high
NPR1	0.890	6.765	1.592	0.235	2.78	Up in ABCC4 high
HIP1	-0.761	7.010	1.685	0.240	2.78	Up in ABCC4 high
KPNA5	-0.736	8.159	1.663	0.204	2.78	Up in ABCC4 high
TRPC3	0.995	6.564	1.422	0.217	2.77	Up in ABCC4 high
GPR116	0.748	9.500	1.681	0.177	2.77	Up in ABCC4 high
ZNF829	-1.180	7.380	1.862	0.252	2.77	Up in ABCC4 high
PLSCR4	1.251	7.500	1.595	0.213	2.77	Up in ABCC4 high
FZD4	-0.481	8.424	1.464	0.174	2.77	Up in ABCC4 high
DCHS1	1.446	7.630	1.570	0.206	2.75	Up in ABCC4 high
A2M	-0.172	7.430	1.617	0.218	2.75	Up in ABCC4 high
NAIP	0.003	7.275	1.687	0.232	2.73	Up in ABCC4 high
KIAA1462	-0.574	7.054	1.567	0.222	2.73	Up in ABCC4 high
ZNF154	0.542	6.663	1.729	0.259	2.72	Up in ABCC4 high
ARHGEF15	-0.271	7.006	1.499	0.214	2.71	Up in ABCC4 high

Supplementary Table 1. Differentially expressed genes between MRP4 high expression carcinomas compared with low MRP4 counterparts.

Gene	Expected score (dExp)	Observed score(d)	Numerator (r)	Denominator (s+s0)	Fold change	Modulation
FAM101B	0.492	6.986	1.439	0.206	2.71	Up in ABCC4 high
PRICKLE2	0.819	7.542	1.755	0.233	2.71	Up in ABCC4 high
PABPC5	-0.001	9.258	2.201	0.238	2.71	Up in ABCC4 high
CCDC102B	0.468	7.361	1.778	0.242	2.71	Up in ABCC4 high
TBCEL	-0.203	9.301	1.722	0.185	2.71	Up in ABCC4 high
ANK2	1.210	7.772	2.577	0.332	2.70	Up in ABCC4 high
LDB2	1.229	8.026	1.743	0.217	2.70	Up in ABCC4 high
CDKL1	0.390	7.857	1.627	0.207	2.69	Up in ABCC4 high
LPHN2	-0.201	7.592	1.619	0.213	2.68	Up in ABCC4 high
NUAK1	0.430	6.701	1.619	0.242	2.68	Up in ABCC4 high
ZDHHC15	-1.137	6.625	1.922	0.290	2.68	Up in ABCC4 high
LPAR1	-0.378	6.521	1.759	0.270	2.68	Up in ABCC4 high
HSPA12B	0.474	7.117	1.495	0.210	2.67	Up in ABCC4 high
ZIK1	-0.072	7.849	1.931	0.246	2.67	Up in ABCC4 high
BCL6B	3.380	7.720	1.447	0.187	2.66	Up in ABCC4 high
MAN1C1	1.500	7.821	1.926	0.246	2.65	Up in ABCC4 high
CBX6	0.566	6.909	1.749	0.253	2.64	Up in ABCC4 high
KCTD20	0.487	9.392	1.449	0.154	2.64	Up in ABCC4 high
LOXHD1	-0.617	6.901	1.376	0.199	2.63	Up in ABCC4 high
ADRBK2	-1.183	6.772	1.595	0.236	2.62	Up in ABCC4 high
CYP2U1	0.032	8.260	1.534	0.186	2.62	Up in ABCC4 high
PRKD3	0.927	8.922	1.475	0.165	2.62	Up in ABCC4 high
ZNF454	0.215	6.753	1.737	0.257	2.61	Up in ABCC4 high
MARCH8	-0.036	7.432	1.559	0.210	2.60	Up in ABCC4 high
ST8SIA4	0.562	7.233	2.012	0.278	2.59	Up in ABCC4 high
ENPEP	0.918	7.204	1.682	0.233	2.58	Up in ABCC4 high
ADAP2	-0.773	7.498	1.396	0.186	2.58	Up in ABCC4 high
MMRN1	0.449	6.766	3.059	0.452	2.57	Up in ABCC4 high
C8orf85	2.880	6.547	1.445	0.221	2.56	Up in ABCC4 high
SEMA5A	-0.215	7.584	1.855	0.245	2.56	Up in ABCC4 high
CCDC75	-1.282	7.464	1.404	0.188	2.56	Up in ABCC4 high
ZMAT3	1.434	6.562	1.413	0.215	2.55	Up in ABCC4 high
PDE2A	1.137	6.815	1.776	0.261	2.54	Up in ABCC4 high
PRR16	-0.111	6.630	1.623	0.245	2.53	Up in ABCC4 high
LATS1	-0.877	6.917	1.565	0.226	2.52	Up in ABCC4 high
OSBPL8	0.086	8.651	1.339	0.155	2.52	Up in ABCC4 high
BMP2K	-0.339	7.237	1.380	0.191	2.52	Up in ABCC4 high
CDYL2	1.760	6.933	1.663	0.240	2.51	Up in ABCC4 high
SGTB	-0.901	9.023	1.473	0.163	2.51	Up in ABCC4 high
DYSF	0.498	7.957	1.485	0.187	2.50	Up in ABCC4 high
RDX	-0.755	7.532	1.920	0.255	2.50	Up in ABCC4 high
C21orf91	-0.238	6.780	1.310	0.193	2.50	Up in ABCC4 high
UBXN7	-0.882	7.037	1.412	0.201	2.50	Up in ABCC4 high
AP1S2	-0.243	8.152	1.652	0.203	2.48	Up in ABCC4 high
PALM2-AKAP2	0.835	8.135	1.407	0.173	2.47	Up in ABCC4 high
CDC14A	0.427	7.491	1.590	0.212	2.47	Up in ABCC4 high
SLC26A2	1.094	10.021	1.392	0.139	2.46	Up in ABCC4 high
FRMD4A	1.199	7.274	1.381	0.190	2.46	Up in ABCC4 high
MYCBP2	-0.007	9.060	1.296	0.143	2.46	Up in ABCC4 high
SASH1	0.051	8.564	1.543	0.180	2.46	Up in ABCC4 high

Supplementary Table 1. Differentially expressed genes between MRP4 high expression carcinomas compared with low MRP4 counterparts.

Gene	Expected score (dExp)	Observed score(d)	Numerator (r)	Denominator (s+s0)	Fold change	Modulation
KLF12	-1.222	7.328	1.436	0.196	2.46	Up in ABCC4 high
MAN1A2	1.852	7.503	1.322	0.176	2.46	Up in ABCC4 high
CSGALNACT2	0.909	7.547	1.334	0.177	2.45	Up in ABCC4 high
TIE1	-0.013	7.273	1.403	0.193	2.45	Up in ABCC4 high
C8orf48	-1.457	7.282	1.646	0.226	2.45	Up in ABCC4 high
RNF217	1.331	6.871	1.951	0.284	2.45	Up in ABCC4 high
DLG2	0.163	7.919	2.650	0.335	2.45	Up in ABCC4 high
ARSB	-0.381	8.464	1.382	0.163	2.44	Up in ABCC4 high
PIK3R1	0.001	7.800	1.356	0.174	2.44	Up in ABCC4 high
ADAMTS18	0.806	6.649	2.326	0.350	2.43	Up in ABCC4 high
WASF3	-0.857	7.958	1.607	0.202	2.43	Up in ABCC4 high
TACC1	-0.474	8.237	1.281	0.155	2.43	Up in ABCC4 high
LRRC8C	-0.513	6.622	1.542	0.233	2.42	Up in ABCC4 high
DSE	0.177	6.780	1.435	0.212	2.42	Up in ABCC4 high
ENTPD1	0.454	9.344	1.282	0.137	2.41	Up in ABCC4 high
DPY19L3	1.497	7.460	1.458	0.195	2.41	Up in ABCC4 high
SLC31A2	0.098	6.990	1.384	0.198	2.41	Up in ABCC4 high
KIAA0922	0.580	7.031	1.674	0.238	2.41	Up in ABCC4 high
STXBP4	-0.388	7.379	1.389	0.188	2.40	Up in ABCC4 high
LNPEP	-1.644	6.694	1.432	0.214	2.40	Up in ABCC4 high
NEK1	-1.039	7.932	1.302	0.164	2.40	Up in ABCC4 high
SH2B3	-0.506	8.805	1.338	0.152	2.38	Up in ABCC4 high
MCC	-0.471	6.907	1.568	0.227	2.38	Up in ABCC4 high
FAM55C	-0.058	7.586	1.598	0.211	2.38	Up in ABCC4 high
MDN1	0.752	7.960	1.472	0.185	2.37	Up in ABCC4 high
CXorf36	-0.055	6.848	1.376	0.201	2.37	Up in ABCC4 high
AKT3	0.450	7.373	1.753	0.238	2.37	Up in ABCC4 high
TTC7B	-1.205	6.862	1.561	0.228	2.37	Up in ABCC4 high
C8orf34	0.515	6.591	1.274	0.193	2.36	Up in ABCC4 high
KDELC1	0.600	6.851	1.298	0.189	2.36	Up in ABCC4 high
ZNF426	0.342	8.075	1.366	0.169	2.36	Up in ABCC4 high
SESN3	0.128	6.748	1.773	0.263	2.35	Up in ABCC4 high
FMNL3	1.315	7.426	1.280	0.172	2.35	Up in ABCC4 high
UHRF1BP1L	-0.069	7.792	1.494	0.192	2.35	Up in ABCC4 high
TECTA	1.034	6.821	1.649	0.242	2.34	Up in ABCC4 high
EEA1	0.718	8.838	1.282	0.145	2.33	Up in ABCC4 high
CTSO	-0.359	7.604	1.244	0.164	2.33	Up in ABCC4 high
HCFC2	0.311	7.506	1.299	0.173	2.33	Up in ABCC4 high
ZNF570	-0.362	7.075	1.572	0.222	2.33	Up in ABCC4 high
ADPRH	0.108	6.755	1.223	0.181	2.32	Up in ABCC4 high
ATP10D	-0.371	7.294	1.364	0.187	2.31	Up in ABCC4 high
C5orf36	1.047	8.249	1.303	0.158	2.31	Up in ABCC4 high
SPG20	-0.839	6.935	1.503	0.217	2.31	Up in ABCC4 high
KLF9	0.935	7.582	1.326	0.175	2.31	Up in ABCC4 high
C9orf102	2.250	6.911	1.370	0.198	2.31	Up in ABCC4 high
HECTD2	-0.156	7.150	1.514	0.212	2.31	Up in ABCC4 high
NRP1	-0.236	7.238	1.405	0.194	2.31	Up in ABCC4 high
FAM126A	1.331	6.618	1.694	0.256	2.30	Up in ABCC4 high
TMEM170B	-1.471	7.011	1.517	0.216	2.30	Up in ABCC4 high
MAP1A	-1.264	6.810	1.534	0.225	2.29	Up in ABCC4 high

Supplementary Table 1. Differentially expressed genes between MRP4 high expression carcinomas compared with low MRP4 counterparts.

Gene	Expected score (dExp)	Observed score(d)	Numerator (r)	Denominator (s+s0)	Fold change	Modulation
APOOL	1.461	7.006	1.420	0.203	2.29	Up in ABCC4 high
MKL2	1.692	7.791	1.154	0.148	2.29	Up in ABCC4 high
PHACTR2	1.235	7.956	1.389	0.175	2.28	Up in ABCC4 high
KIAA1432	0.489	8.863	1.207	0.136	2.28	Up in ABCC4 high
PRKCH	-0.372	6.597	1.288	0.195	2.28	Up in ABCC4 high
LIPA	1.051	6.744	1.088	0.161	2.27	Up in ABCC4 high
FBXO30	-1.101	9.485	1.223	0.129	2.27	Up in ABCC4 high
CD34	0.777	6.847	1.274	0.186	2.27	Up in ABCC4 high
LAMB1	-1.041	8.626	1.341	0.156	2.26	Up in ABCC4 high
ATM	0.058	6.711	1.323	0.197	2.26	Up in ABCC4 high
KLHL8	1.426	7.729	1.251	0.162	2.24	Up in ABCC4 high
CEP97	-0.587	6.514	1.389	0.213	2.24	Up in ABCC4 high
FOXN3	-1.509	8.974	1.182	0.132	2.24	Up in ABCC4 high
DENND5A	0.003	8.403	1.294	0.154	2.22	Up in ABCC4 high
ZNF221	-0.562	7.768	1.283	0.165	2.21	Up in ABCC4 high
ZNF788	0.117	7.701	1.523	0.198	2.21	Up in ABCC4 high
STARD13	-0.294	8.119	1.234	0.152	2.21	Up in ABCC4 high
MMD	-0.422	6.571	1.266	0.193	2.19	Up in ABCC4 high
ZNF641	-0.772	7.552	1.302	0.172	2.19	Up in ABCC4 high
MANEA	1.672	7.117	1.268	0.178	2.17	Up in ABCC4 high
MED13L	-1.256	8.016	1.209	0.151	2.17	Up in ABCC4 high
PIK3CA	-0.647	6.856	1.193	0.174	2.17	Up in ABCC4 high
FAM171B	0.441	7.078	1.915	0.271	2.17	Up in ABCC4 high
KIAA1586	-0.776	9.996	1.213	0.121	2.16	Up in ABCC4 high
SOAT1	0.344	6.598	1.216	0.184	2.16	Up in ABCC4 high
GCLM	1.489	7.124	1.198	0.168	2.16	Up in ABCC4 high
SNX18	0.614	10.316	1.170	0.113	2.16	Up in ABCC4 high
FGFR1	-1.376	6.522	1.368	0.210	2.16	Up in ABCC4 high
PRCP	-0.752	9.031	1.151	0.127	2.15	Up in ABCC4 high
VASH1	-0.437	7.063	1.385	0.196	2.15	Up in ABCC4 high
MEX3B	0.807	6.605	1.257	0.190	2.15	Up in ABCC4 high
AFAP1L1	0.646	6.870	1.355	0.197	2.13	Up in ABCC4 high
ZFX	0.776	7.786	1.220	0.157	2.13	Up in ABCC4 high
RECQL	-1.176	7.175	1.214	0.169	2.13	Up in ABCC4 high
C5orf42	-0.596	7.013	1.231	0.176	2.13	Up in ABCC4 high
EHD3	0.656	7.363	1.432	0.194	2.12	Up in ABCC4 high
OSTM1	-2.003	10.059	1.142	0.114	2.12	Up in ABCC4 high
ATG4C	-2.151	8.003	1.141	0.143	2.12	Up in ABCC4 high
SATB1	0.083	6.928	1.135	0.164	2.12	Up in ABCC4 high
ZBTB38	-0.906	7.356	1.152	0.157	2.11	Up in ABCC4 high
CEP170	-0.068	6.652	1.350	0.203	2.10	Up in ABCC4 high
TRIO	1.800	7.512	1.266	0.168	2.10	Up in ABCC4 high
ZNF420	0.342	6.698	1.312	0.196	2.10	Up in ABCC4 high
MTMR9	-0.407	8.478	1.079	0.127	2.09	Up in ABCC4 high
ACVRL1	-0.301	7.324	1.081	0.148	2.09	Up in ABCC4 high
ERCC4	0.577	8.898	1.159	0.130	2.09	Up in ABCC4 high
SESN1	0.705	6.699	1.166	0.174	2.09	Up in ABCC4 high
KLHL5	1.424	7.808	1.420	0.182	2.09	Up in ABCC4 high
NEDD4	0.347	6.725	1.234	0.184	2.09	Up in ABCC4 high
NLGN4X	1.701	7.258	2.651	0.365	2.08	Up in ABCC4 high

Supplementary Table 1. Differentially expressed genes between MRP4 high expression carcinomas compared with low MRP4 counterparts.

Gene	Expected score (dExp)	Observed score(d)	Numerator (r)	Denominator (s+s0)	Fold change	Modulation
JAK2	0.894	6.981	1.182	0.169	2.07	Up in ABCC4 high
RPL23AP53	1.223	8.352	1.081	0.129	2.07	Up in ABCC4 high
APC	-2.040	7.969	1.105	0.139	2.05	Up in ABCC4 high
CHD9	-1.592	7.878	1.080	0.137	2.05	Up in ABCC4 high
HERC1	-0.034	8.234	1.107	0.134	2.04	Up in ABCC4 high
RAB11FIP2	0.166	9.271	1.024	0.110	2.04	Up in ABCC4 high
ZFP106	1.848	8.094	1.088	0.134	2.04	Up in ABCC4 high
PIKFYVE	-1.585	7.641	1.107	0.145	2.04	Up in ABCC4 high
TWSG1	0.068	7.078	1.114	0.157	2.04	Up in ABCC4 high
KATNAL1	0.251	6.914	1.365	0.197	2.04	Up in ABCC4 high
RHOQ	-1.031	8.194	1.037	0.127	2.04	Up in ABCC4 high
KAT2B	-0.924	7.205	1.018	0.141	2.03	Up in ABCC4 high
ZHX3	-0.958	7.345	1.081	0.147	2.03	Up in ABCC4 high
ATF2	-0.459	6.647	1.253	0.189	2.02	Up in ABCC4 high
HSPA13	1.134	6.695	1.135	0.169	2.02	Up in ABCC4 high
SETD7	1.016	7.380	1.036	0.140	2.02	Up in ABCC4 high
MAP3K3	1.009	8.532	1.174	0.138	2.02	Up in ABCC4 high
TRIM23	-0.545	7.763	1.130	0.146	2.01	Up in ABCC4 high
VPS13B	-1.517	8.381	1.084	0.129	2.01	Up in ABCC4 high
TMTC3	0.621	9.388	1.044	0.111	2.01	Up in ABCC4 high
DENND4A	-2.521	7.421	1.068	0.144	2.01	Up in ABCC4 high
FAM3A	-1.291	-7.809	-1.027	0.132	0.50	Down in ABCC4 high
ANKZF1	-0.474	-7.653	-1.045	0.137	0.49	Down in ABCC4 high
TRPT1	-1.268	-10.537	-1.000	0.095	0.49	Down in ABCC4 high
FAM98C	-0.179	-7.640	-0.971	0.127	0.49	Down in ABCC4 high
POLR2H	-0.774	-8.152	-0.946	0.116	0.49	Down in ABCC4 high
MPG	-1.099	-7.567	-1.020	0.135	0.49	Down in ABCC4 high
EML2	-0.045	-8.001	-0.968	0.121	0.48	Down in ABCC4 high
MRPS34	-0.938	-7.592	-0.996	0.131	0.47	Down in ABCC4 high
OCEL1	-0.243	-7.665	-1.004	0.131	0.47	Down in ABCC4 high
DEDD2	-0.507	-7.568	-1.074	0.142	0.47	Down in ABCC4 high
SH3GLB2	0.241	-7.656	-1.086	0.142	0.47	Down in ABCC4 high
PNKP	-0.546	-8.250	-1.106	0.134	0.47	Down in ABCC4 high
TMEM134	1.220	-8.485	-1.110	0.131	0.46	Down in ABCC4 high
RAD9A	-1.836	-8.317	-1.122	0.135	0.46	Down in ABCC4 high
BOLA2	1.303	-7.458	-1.021	0.137	0.46	Down in ABCC4 high
CDC34	-1.094	-7.536	-1.083	0.144	0.46	Down in ABCC4 high
MRPL52	0.324	-7.862	-1.085	0.138	0.46	Down in ABCC4 high
LSM4	-0.491	-8.822	-1.090	0.124	0.46	Down in ABCC4 high
ALDOA	2.046	-8.468	-1.166	0.138	0.45	Down in ABCC4 high
ZNF511	-0.261	-9.338	-1.089	0.117	0.45	Down in ABCC4 high
PNPLA2	0.248	-7.478	-1.141	0.153	0.45	Down in ABCC4 high
TRAF4	0.878	-7.746	-1.129	0.146	0.44	Down in ABCC4 high
RNF126	-0.414	-8.670	-1.269	0.146	0.43	Down in ABCC4 high
NDOR1	-0.566	-7.909	-1.207	0.153	0.43	Down in ABCC4 high
MGC70857	-1.664	-8.128	-1.177	0.145	0.42	Down in ABCC4 high
DGAT1	0.033	-7.958	-1.222	0.154	0.41	Down in ABCC4 high
TIAF1	-1.161	-7.893	-1.199	0.152	0.41	Down in ABCC4 high
TMUB1	-0.800	-7.831	-1.386	0.177	0.41	Down in ABCC4 high
ACP6	-0.434	-7.819	-1.232	0.158	0.41	Down in ABCC4 high

Supplementary Table 1. Differentially expressed genes between MRP4 high expression carcinomas compared with low MRP4 counterparts.

Gene	Expected score (dExp)	Observed score(d)	Numerator (r)	Denominator (s+s0)	Fold change	Modulation
DCI	-0.549	-7.626	-1.240	0.163	0.41	Down in ABCC4 high
NT5C	0.235	-7.491	-1.312	0.175	0.41	Down in ABCC4 high
EBP	-1.646	-8.424	-1.271	0.151	0.40	Down in ABCC4 high
IGSF8	-0.092	-7.850	-1.213	0.155	0.40	Down in ABCC4 high
YDJC	1.309	-7.461	-1.362	0.183	0.39	Down in ABCC4 high
EXD3	-0.914	-7.789	-1.449	0.186	0.38	Down in ABCC4 high
NR2F6	0.919	-10.239	-1.436	0.140	0.37	Down in ABCC4 high
TMEM141	-1.913	-10.618	-1.465	0.138	0.37	Down in ABCC4 high
C7orf47	0.641	-8.956	-1.508	0.168	0.36	Down in ABCC4 high
ABHD11	0.136	-7.816	-1.416	0.181	0.36	Down in ABCC4 high
TIMM16	-0.107	-7.932	-1.396	0.176	0.36	Down in ABCC4 high
GIYD2	0.538	-7.921	-1.454	0.184	0.35	Down in ABCC4 high
SAMD10	0.164	-8.915	-1.545	0.173	0.34	Down in ABCC4 high
ARRDC1	-0.593	-7.919	-1.500	0.189	0.34	Down in ABCC4 high
STARD10	-0.223	-8.178	-1.494	0.183	0.34	Down in ABCC4 high
TSTD1	1.161	-8.236	-1.496	0.182	0.33	Down in ABCC4 high
PPDPF	0.977	-10.055	-1.860	0.185	0.30	Down in ABCC4 high
BBC3	1.772	-7.431	-1.780	0.240	0.29	Down in ABCC4 high
C11orf35	-1.495	-7.565	-2.040	0.270	0.28	Down in ABCC4 high
RASSF7	-1.600	-8.649	-2.117	0.245	0.27	Down in ABCC4 high

Supplementary Table 2. Total down- and upregulated transcripts in PANC1-MRP4sh cells compared to PANC1-scramble cells.

Gene	logFC	logCPM	PValue	FDR	Modulation
COL4A5	-7.486	0.827	8.421E-07	1.216E-04	Down in PANC1-MRP4sh
GATA5	-5.263	3.078	3.044E-08	6.860E-06	Down in PANC1-MRP4sh
DSP	-4.611	1.112	3.888E-07	6.488E-05	Down in PANC1-MRP4sh
C20orf166-AS1	-4.210	2.646	8.538E-05	6.268E-03	Down in PANC1-MRP4sh
KRT19	-4.164	4.734	5.304E-14	4.259E-11	Down in PANC1-MRP4sh
LIMCH1	-3.980	1.071	1.311E-06	1.719E-04	Down in PANC1-MRP4sh
COL6A2	-3.963	2.420	1.128E-07	2.196E-05	Down in PANC1-MRP4sh
ADGRB1	-3.842	0.951	1.263E-06	1.672E-04	Down in PANC1-MRP4sh
ELFN1	-3.748	4.244	1.142E-25	2.933E-22	Down in PANC1-MRP4sh
SEMA3F	-3.581	1.355	7.986E-07	1.166E-04	Down in PANC1-MRP4sh
EFNB2	-3.538	1.047	4.966E-06	5.596E-04	Down in PANC1-MRP4sh
RPS6KA2	-3.490	2.902	2.709E-05	2.384E-03	Down in PANC1-MRP4sh
AMOT	-3.476	1.997	3.126E-07	5.428E-05	Down in PANC1-MRP4sh
HAS2-AS1	-3.326	0.556	1.869E-04	1.117E-02	Down in PANC1-MRP4sh
NES	-3.310	2.441	6.620E-06	7.207E-04	Down in PANC1-MRP4sh
ONECUT3	-3.310	2.143	8.606E-08	1.755E-05	Down in PANC1-MRP4sh
GABBR2	-3.299	3.170	8.248E-06	8.837E-04	Down in PANC1-MRP4sh
DMKN	-3.234	1.457	2.095E-04	1.198E-02	Down in PANC1-MRP4sh
H19	-3.068	4.337	7.167E-07	1.058E-04	Down in PANC1-MRP4sh
FSTL1	-3.009	1.863	3.216E-05	2.755E-03	Down in PANC1-MRP4sh
CRLF1	-2.915	0.254	1.066E-03	4.237E-02	Down in PANC1-MRP4sh
MARCH4	-2.911	3.254	1.497E-12	7.695E-10	Down in PANC1-MRP4sh
GABARAPL1	-2.859	2.318	6.610E-05	5.055E-03	Down in PANC1-MRP4sh
NEU4	-2.842	0.519	9.730E-04	3.993E-02	Down in PANC1-MRP4sh
TXNIP	-2.781	1.559	5.997E-05	4.756E-03	Down in PANC1-MRP4sh
WT1	-2.672	1.356	3.070E-05	2.647E-03	Down in PANC1-MRP4sh
CES1	-2.662	3.752	1.315E-13	9.940E-11	Down in PANC1-MRP4sh
KRT15	-2.656	1.596	5.400E-06	5.930E-04	Down in PANC1-MRP4sh
IGFBP2	-2.645	1.913	6.036E-05	4.758E-03	Down in PANC1-MRP4sh
COL5A2	-2.621	1.306	8.840E-05	6.380E-03	Down in PANC1-MRP4sh
CLDN10	-2.617	2.300	1.568E-05	1.515E-03	Down in PANC1-MRP4sh
HAPLN3	-2.547	3.933	4.334E-04	2.141E-02	Down in PANC1-MRP4sh
AATK	-2.519	1.081	2.056E-04	1.195E-02	Down in PANC1-MRP4sh
WNK2	-2.517	1.591	1.403E-04	8.970E-03	Down in PANC1-MRP4sh
HOXA2	-2.498	1.073	1.592E-04	9.812E-03	Down in PANC1-MRP4sh
PCDH7	-2.490	0.721	6.049E-04	2.795E-02	Down in PANC1-MRP4sh
FAM65C	-2.478	3.167	4.361E-09	1.245E-06	Down in PANC1-MRP4sh
PLXND1	-2.420	6.434	4.836E-25	1.035E-21	Down in PANC1-MRP4sh
CPPED1	-2.408	1.502	3.166E-04	1.660E-02	Down in PANC1-MRP4sh
FAM132A	-2.396	0.998	4.598E-04	2.229E-02	Down in PANC1-MRP4sh
SHANK2	-2.390	2.106	1.368E-06	1.775E-04	Down in PANC1-MRP4sh
GDA	-2.355	0.957	1.329E-03	4.977E-02	Down in PANC1-MRP4sh
HAS2	-2.330	1.559	4.951E-05	4.052E-03	Down in PANC1-MRP4sh
DACT2	-2.176	5.172	1.712E-17	2.524E-14	Down in PANC1-MRP4sh
HAP1	-2.144	1.320	5.998E-04	2.792E-02	Down in PANC1-MRP4sh
MFGE8	-2.105	7.494	6.402E-05	4.981E-03	Down in PANC1-MRP4sh
KIF1A	-2.104	5.097	3.575E-12	1.701E-09	Down in PANC1-MRP4sh
GLB1L2	-2.034	3.212	2.317E-07	4.134E-05	Down in PANC1-MRP4sh
CYB5R2	-2.016	4.360	1.079E-04	7.504E-03	Down in PANC1-MRP4sh
LOC643201	-2.009	3.944	9.567E-06	1.007E-03	Down in PANC1-MRP4sh
CD22	-1.989	1.313	1.316E-03	4.943E-02	Down in PANC1-MRP4sh
PELI1	-1.985	1.934	1.387E-04	8.907E-03	Down in PANC1-MRP4sh
COL5A1	-1.966	1.791	5.924E-04	2.768E-02	Down in PANC1-MRP4sh
FAM107B	-1.943	5.752	2.230E-04	1.259E-02	Down in PANC1-MRP4sh
EFHD1	-1.874	2.278	1.295E-04	8.489E-03	Down in PANC1-MRP4sh
ID2	-1.873	3.731	1.173E-05	1.215E-03	Down in PANC1-MRP4sh

Supplementary Table 2. Total down- and upregulated transcripts in PANC1-MRP4sh cells compared to PANC1-scramble cells.

Gene	logFC	logCPM	PValue	FDR	Modulation
MARVELD2	-1.853	1.558	7.065E-04	3.141E-02	Down in PANC1-MRP4sh
MXRA8	-1.848	5.418	3.017E-06	3.523E-04	Down in PANC1-MRP4sh
KCNQ2	-1.842	2.111	1.157E-04	7.863E-03	Down in PANC1-MRP4sh
UNC5C	-1.823	2.417	8.698E-05	6.313E-03	Down in PANC1-MRP4sh
ZNF503	-1.792	3.186	1.777E-04	1.072E-02	Down in PANC1-MRP4sh
ABLIM1	-1.790	4.348	1.101E-04	7.562E-03	Down in PANC1-MRP4sh
MAP7	-1.750	2.827	2.384E-04	1.326E-02	Down in PANC1-MRP4sh
HOXA3	-1.721	3.079	4.755E-05	3.916E-03	Down in PANC1-MRP4sh
TP53I11	-1.680	5.580	1.462E-05	1.444E-03	Down in PANC1-MRP4sh
IL1R1	-1.670	2.604	2.177E-04	1.237E-02	Down in PANC1-MRP4sh
MARCKS	-1.653	5.063	1.769E-05	1.683E-03	Down in PANC1-MRP4sh
DHRS3	-1.651	3.877	4.881E-06	5.549E-04	Down in PANC1-MRP4sh
CACNA1H	-1.643	4.989	2.312E-05	2.107E-03	Down in PANC1-MRP4sh
MEG3	-1.623	5.126	6.026E-04	2.795E-02	Down in PANC1-MRP4sh
HAGLR	-1.605	2.412	1.259E-03	4.785E-02	Down in PANC1-MRP4sh
MDK	-1.592	6.474	2.277E-08	5.520E-06	Down in PANC1-MRP4sh
ZNF608	-1.573	2.917	9.173E-05	6.583E-03	Down in PANC1-MRP4sh
GAL	-1.572	4.173	2.395E-05	2.152E-03	Down in PANC1-MRP4sh
ZNF385D	-1.564	3.059	2.994E-04	1.588E-02	Down in PANC1-MRP4sh
CACNA1G	-1.512	2.558	4.731E-04	2.276E-02	Down in PANC1-MRP4sh
FLNC	-1.494	3.331	7.107E-04	3.148E-02	Down in PANC1-MRP4sh
**ABCC4**	**-1.486**	**4.613**	**4.961E-07**	**7.679E-05**	**Down in PANC1-MRP4sh**
ALDH1A3	-1.485	3.880	1.207E-05	1.240E-03	Down in PANC1-MRP4sh
NKX6-2	-1.469	5.556	1.116E-07	2.196E-05	Down in PANC1-MRP4sh
SLC7A11	-1.464	3.585	5.013E-05	4.076E-03	Down in PANC1-MRP4sh
LAMC3	-1.463	4.628	1.198E-07	2.296E-05	Down in PANC1-MRP4sh
SH3BP2	-1.453	4.099	1.782E-06	2.223E-04	Down in PANC1-MRP4sh
PCOLCE2	-1.449	3.106	2.013E-04	1.181E-02	Down in PANC1-MRP4sh
SESTD1	-1.446	2.512	7.803E-04	3.375E-02	Down in PANC1-MRP4sh
NA	-1.442	4.421	1.385E-04	8.907E-03	Down in PANC1-MRP4sh
CABLES1	-1.432	5.049	3.725E-07	6.297E-05	Down in PANC1-MRP4sh
SLC47A1	-1.423	2.677	6.213E-04	2.831E-02	Down in PANC1-MRP4sh
CPLX1	-1.416	2.834	9.899E-04	4.050E-02	Down in PANC1-MRP4sh
NRARP	-1.403	4.299	1.600E-06	2.035E-04	Down in PANC1-MRP4sh
OCLN	-1.399	3.658	1.090E-04	7.531E-03	Down in PANC1-MRP4sh
SESN2	-1.314	4.001	8.911E-04	3.741E-02	Down in PANC1-MRP4sh
PHGDH	-1.294	7.492	1.281E-05	1.295E-03	Down in PANC1-MRP4sh
CLDN7	-1.285	3.414	3.413E-04	1.754E-02	Down in PANC1-MRP4sh
ADGRL2	-1.272	4.955	6.859E-04	3.103E-02	Down in PANC1-MRP4sh
LGR4	-1.260	4.320	3.357E-04	1.746E-02	Down in PANC1-MRP4sh
MAGI2-AS3	-1.235	4.153	5.340E-05	4.315E-03	Down in PANC1-MRP4sh
JAG2	-1.233	4.200	7.547E-05	5.703E-03	Down in PANC1-MRP4sh
LYN	-1.206	3.467	1.107E-03	4.316E-02	Down in PANC1-MRP4sh
EGFL7	-1.144	3.875	8.263E-04	3.515E-02	Down in PANC1-MRP4sh
SLC7A5	-1.117	8.393	2.160E-06	2.618E-04	Down in PANC1-MRP4sh
ESRP2	-1.056	4.765	4.561E-04	2.219E-02	Down in PANC1-MRP4sh
VWA1	-1.052	6.807	1.690E-05	1.620E-03	Down in PANC1-MRP4sh
F13A1	6.670	3.633	3.134E-26	1.007E-22	Up in PANC1-MRP4sh
PRICKLE2-AS1	6.228	-0.089	5.879E-05	4.721E-03	Up in PANC1-MRP4sh
PRICKLE2	5.425	1.173	4.732E-09	1.322E-06	Up in PANC1-MRP4sh
PLPP4	4.899	0.739	9.304E-07	1.328E-04	Up in PANC1-MRP4sh
RASL10A	4.260	5.061	6.936E-34	8.910E-30	Up in PANC1-MRP4sh
NMBR	4.138	0.156	1.544E-04	9.674E-03	Up in PANC1-MRP4sh
TRIP6	4.084	3.451	6.343E-18	1.164E-14	Up in PANC1-MRP4sh
ZDHHC15	4.008	0.063	3.413E-04	1.754E-02	Up in PANC1-MRP4sh
SUSD4	3.920	1.472	1.087E-08	2.909E-06	Up in PANC1-MRP4sh

Supplementary Table 2. Total down- and upregulated transcripts in PANC1-MRP4sh cells compared to PANC1-scramble cells.

Gene	logFC	logCPM	PValue	FDR	Modulation
MN1	3.881	2.316	5.120E-11	2.268E-08	Up in PANC1-MRP4sh
SLC37A2	3.792	-0.088	7.006E-04	3.136E-02	Up in PANC1-MRP4sh
RRAD	3.703	2.166	8.922E-10	3.184E-07	Up in PANC1-MRP4sh
PRSS12	3.660	3.222	1.121E-15	1.107E-12	Up in PANC1-MRP4sh
NIPAL4	3.620	1.914	1.673E-09	5.372E-07	Up in PANC1-MRP4sh
RGS4	3.421	5.508	1.702E-14	1.562E-11	Up in PANC1-MRP4sh
PCLO	3.283	1.643	4.906E-07	7.679E-05	Up in PANC1-MRP4sh
GPR173	3.227	0.156	8.979E-04	3.757E-02	Up in PANC1-MRP4sh
STK33	3.112	2.077	2.571E-08	6.005E-06	Up in PANC1-MRP4sh
CDS1	2.885	1.628	2.641E-06	3.112E-04	Up in PANC1-MRP4sh
IL11	2.847	6.045	2.441E-30	1.568E-26	Up in PANC1-MRP4sh
ST3GAL5	2.754	6.194	2.709E-26	1.007E-22	Up in PANC1-MRP4sh
MAMLD1	2.741	6.176	7.455E-17	8.707E-14	Up in PANC1-MRP4sh
BMF	2.689	4.649	3.436E-17	4.414E-14	Up in PANC1-MRP4sh
CDH17	2.679	1.435	1.902E-04	1.128E-02	Up in PANC1-MRP4sh
CX3CL1	2.649	5.223	2.316E-06	2.780E-04	Up in PANC1-MRP4sh
NEBL	2.638	3.618	1.615E-12	7.981E-10	Up in PANC1-MRP4sh
IGSF9	2.629	1.108	8.141E-05	6.071E-03	Up in PANC1-MRP4sh
CADM1	2.587	5.274	3.674E-13	2.622E-10	Up in PANC1-MRP4sh
VSTM2L	2.576	3.588	3.859E-10	1.599E-07	Up in PANC1-MRP4sh
TRO	2.558	0.884	2.831E-04	1.534E-02	Up in PANC1-MRP4sh
MYLK	2.496	6.699	5.631E-13	3.617E-10	Up in PANC1-MRP4sh
LRRC36	2.484	0.616	1.034E-03	4.191E-02	Up in PANC1-MRP4sh
SPTB	2.478	5.581	2.335E-16	2.500E-13	Up in PANC1-MRP4sh
TMEM92	2.420	2.238	3.878E-06	4.489E-04	Up in PANC1-MRP4sh
PLA2R1	2.404	2.164	2.613E-05	2.315E-03	Up in PANC1-MRP4sh
DMD	2.376	2.783	4.271E-07	6.946E-05	Up in PANC1-MRP4sh
DOCK2	2.320	1.201	7.056E-04	3.141E-02	Up in PANC1-MRP4sh
CPS1	2.279	3.359	4.111E-06	4.715E-04	Up in PANC1-MRP4sh
ZHX2	2.263	1.422	1.016E-03	4.145E-02	Up in PANC1-MRP4sh
SLC2A12	2.233	4.170	4.655E-10	1.812E-07	Up in PANC1-MRP4sh
ANO9	2.195	1.455	1.267E-04	8.389E-03	Up in PANC1-MRP4sh
CPA4	2.192	3.710	1.338E-09	4.408E-07	Up in PANC1-MRP4sh
PTGES3L	2.176	1.645	1.592E-04	9.812E-03	Up in PANC1-MRP4sh
MIR147B	2.175	3.723	7.281E-08	1.533E-05	Up in PANC1-MRP4sh
NDP	2.151	1.418	1.709E-04	1.035E-02	Up in PANC1-MRP4sh
KSR2	2.148	2.136	5.950E-05	4.748E-03	Up in PANC1-MRP4sh
TPPP3	2.111	1.869	4.496E-04	2.213E-02	Up in PANC1-MRP4sh
AK5	2.090	5.095	1.197E-08	3.140E-06	Up in PANC1-MRP4sh
LIPG	2.082	4.240	1.140E-09	3.854E-07	Up in PANC1-MRP4sh
MGLL	2.052	5.450	4.019E-07	6.620E-05	Up in PANC1-MRP4sh
CCL2	2.034	5.757	2.452E-09	7.501E-07	Up in PANC1-MRP4sh
OR7E14P	2.029	1.798	2.677E-04	1.457E-02	Up in PANC1-MRP4sh
TLN2	2.002	5.277	1.871E-05	1.767E-03	Up in PANC1-MRP4sh
CST1	1.978	1.679	6.096E-04	2.807E-02	Up in PANC1-MRP4sh
COL7A1	1.973	5.441	8.051E-13	4.309E-10	Up in PANC1-MRP4sh
C15orf48	1.968	4.959	7.955E-13	4.309E-10	Up in PANC1-MRP4sh
KSR1	1.958	4.216	1.122E-05	1.172E-03	Up in PANC1-MRP4sh
MYLK-AS1	1.919	2.633	9.487E-06	1.007E-03	Up in PANC1-MRP4sh
IFT46	1.914	2.092	1.162E-04	7.863E-03	Up in PANC1-MRP4sh
CD82	1.890	6.073	1.768E-17	2.524E-14	Up in PANC1-MRP4sh
WNT5B	1.888	5.941	5.146E-10	1.945E-07	Up in PANC1-MRP4sh
HTRA1	1.872	4.469	6.323E-10	2.321E-07	Up in PANC1-MRP4sh
KALRN	1.863	2.592	1.971E-05	1.822E-03	Up in PANC1-MRP4sh
NKAIN4	1.857	2.162	1.906E-04	1.128E-02	Up in PANC1-MRP4sh
GPR176	1.856	3.709	2.113E-05	1.939E-03	Up in PANC1-MRP4sh

Supplementary Table 2. Total down- and upregulated transcripts in PANC1-MRP4sh cells compared to PANC1-scramble cells.

Gene	logFC	logCPM	PValue	FDR	Modulation
OLFML2B	1.849	4.698	1.321E-08	3.393E-06	Up in PANC1-MRP4sh
PPP2R3A	1.792	3.426	1.672E-06	2.106E-04	Up in PANC1-MRP4sh
MARCH9	1.775	3.332	1.163E-04	7.863E-03	Up in PANC1-MRP4sh
PPP1R3C	1.771	2.105	1.993E-04	1.174E-02	Up in PANC1-MRP4sh
MDGA1	1.751	4.155	1.954E-05	1.819E-03	Up in PANC1-MRP4sh
BPGM	1.720	6.988	2.239E-09	7.015E-07	Up in PANC1-MRP4sh
IL32	1.720	4.729	1.049E-09	3.641E-07	Up in PANC1-MRP4sh
SFRP1	1.718	7.065	2.226E-14	1.906E-11	Up in PANC1-MRP4sh
SGK1	1.704	4.749	8.551E-09	2.337E-06	Up in PANC1-MRP4sh
LONRF3	1.703	2.340	2.099E-04	1.198E-02	Up in PANC1-MRP4sh
TRIB1	1.702	5.683	8.385E-11	3.591E-08	Up in PANC1-MRP4sh
ARHGAP5-AS1	1.692	2.060	1.079E-03	4.237E-02	Up in PANC1-MRP4sh
TRHDE	1.691	4.106	1.236E-06	1.653E-04	Up in PANC1-MRP4sh
NUAK2	1.658	4.974	2.235E-04	1.259E-02	Up in PANC1-MRP4sh
ARMCX1	1.652	2.130	6.887E-04	3.104E-02	Up in PANC1-MRP4sh
INHBB	1.642	7.828	6.594E-13	3.851E-10	Up in PANC1-MRP4sh
KRT81	1.598	3.051	2.075E-04	1.198E-02	Up in PANC1-MRP4sh
DSC2	1.594	5.052	4.304E-10	1.728E-07	Up in PANC1-MRP4sh
LTBP1	1.585	7.245	6.974E-12	3.200E-09	Up in PANC1-MRP4sh
EBI3	1.577	2.162	7.923E-04	3.415E-02	Up in PANC1-MRP4sh
MEST	1.571	3.778	1.207E-04	8.067E-03	Up in PANC1-MRP4sh
PLAUR	1.553	3.639	1.435E-05	1.440E-03	Up in PANC1-MRP4sh
SLC41A2	1.536	2.351	5.375E-04	2.539E-02	Up in PANC1-MRP4sh
SLC39A8	1.528	2.130	1.065E-03	4.237E-02	Up in PANC1-MRP4sh
VEGFA	1.509	7.131	2.799E-05	2.447E-03	Up in PANC1-MRP4sh
NANOS1	1.508	4.727	7.045E-08	1.508E-05	Up in PANC1-MRP4sh
AGPAT3	1.505	5.455	9.928E-07	1.402E-04	Up in PANC1-MRP4sh
TRHDE-AS1	1.501	2.992	7.477E-04	3.278E-02	Up in PANC1-MRP4sh
KRT7	1.483	3.465	2.583E-04	1.418E-02	Up in PANC1-MRP4sh
NPTX1	1.481	3.759	9.610E-05	6.859E-03	Up in PANC1-MRP4sh
HTR1D	1.468	4.705	1.064E-06	1.486E-04	Up in PANC1-MRP4sh
IGFBP4	1.466	11.430	4.772E-13	3.227E-10	Up in PANC1-MRP4sh
PXYLP1	1.463	2.753	1.173E-03	4.514E-02	Up in PANC1-MRP4sh
PON3	1.460	4.130	1.496E-06	1.922E-04	Up in PANC1-MRP4sh
DPYD	1.445	2.909	1.271E-03	4.818E-02	Up in PANC1-MRP4sh
CDCP1	1.444	8.248	6.491E-13	3.851E-10	Up in PANC1-MRP4sh
ADGRG1	1.440	5.995	1.042E-03	4.195E-02	Up in PANC1-MRP4sh
LXN	1.423	6.280	1.521E-07	2.792E-05	Up in PANC1-MRP4sh
SLC16A2	1.422	5.420	1.266E-05	1.291E-03	Up in PANC1-MRP4sh
GRB10	1.420	7.947	5.195E-07	7.852E-05	Up in PANC1-MRP4sh
ZNF275	1.417	3.496	6.678E-05	5.077E-03	Up in PANC1-MRP4sh
KRT80	1.410	7.360	6.518E-05	5.014E-03	Up in PANC1-MRP4sh
NRP2	1.392	5.378	5.105E-06	5.703E-04	Up in PANC1-MRP4sh
CYTH4	1.384	3.500	1.081E-04	7.504E-03	Up in PANC1-MRP4sh
SIPA1L2	1.380	3.994	1.206E-04	8.067E-03	Up in PANC1-MRP4sh
SPRED3	1.359	2.948	1.058E-03	4.233E-02	Up in PANC1-MRP4sh
SIK1	1.337	6.144	3.961E-09	1.156E-06	Up in PANC1-MRP4sh
IQSEC2	1.333	4.274	3.285E-05	2.795E-03	Up in PANC1-MRP4sh
KIAA1522	1.327	5.522	2.680E-07	4.716E-05	Up in PANC1-MRP4sh
CDC42EP3	1.317	4.041	8.780E-04	3.698E-02	Up in PANC1-MRP4sh
TCEAL3	1.297	5.425	4.242E-08	9.395E-06	Up in PANC1-MRP4sh
MOCS1	1.297	5.121	8.156E-08	1.690E-05	Up in PANC1-MRP4sh
EFR3B	1.296	6.139	2.907E-09	8.684E-07	Up in PANC1-MRP4sh
GLCE	1.296	4.819	1.544E-05	1.503E-03	Up in PANC1-MRP4sh
EREG	1.292	4.554	4.588E-05	3.802E-03	Up in PANC1-MRP4sh
TRPV2	1.291	5.519	2.109E-08	5.314E-06	Up in PANC1-MRP4sh

Supplementary Table 2. Total down- and upregulated transcripts in PANC1-MRP4sh cells compared to PANC1-scramble cells.

Gene	logFC	logCPM	PValue	FDR	Modulation
PTPRU	1.273	4.994	2.374E-05	2.148E-03	Up in PANC1-MRP4sh
STAC	1.273	5.605	1.113E-06	1.537E-04	Up in PANC1-MRP4sh
GALNT6	1.269	3.266	1.072E-03	4.237E-02	Up in PANC1-MRP4sh
NFKB2	1.265	6.667	5.124E-07	7.837E-05	Up in PANC1-MRP4sh
AKAP2	1.264	6.207	2.181E-08	5.387E-06	Up in PANC1-MRP4sh
PALM2-AKAP2	1.256	6.206	2.825E-08	6.481E-06	Up in PANC1-MRP4sh
GJD3	1.241	4.940	1.458E-05	1.444E-03	Up in PANC1-MRP4sh
PLEKHA7	1.241	4.089	8.625E-04	3.645E-02	Up in PANC1-MRP4sh
TSPAN6	1.238	5.349	1.272E-07	2.369E-05	Up in PANC1-MRP4sh
KCNK6	1.232	4.977	1.183E-06	1.617E-04	Up in PANC1-MRP4sh
GPRASP2	1.220	4.169	4.542E-05	3.789E-03	Up in PANC1-MRP4sh
DTX3L	1.211	3.971	2.866E-04	1.547E-02	Up in PANC1-MRP4sh
DKK3	1.203	7.798	1.698E-07	3.072E-05	Up in PANC1-MRP4sh
AHR	1.200	5.264	3.654E-07	6.259E-05	Up in PANC1-MRP4sh
TRAFD1	1.193	6.149	2.495E-08	5.935E-06	Up in PANC1-MRP4sh
BIRC3	1.190	5.995	2.918E-05	2.533E-03	Up in PANC1-MRP4sh
LAMB1	1.183	7.241	5.846E-07	8.734E-05	Up in PANC1-MRP4sh
PLEKHG4	1.179	4.238	2.997E-04	1.588E-02	Up in PANC1-MRP4sh
NTNG2	1.178	5.787	4.904E-07	7.679E-05	Up in PANC1-MRP4sh
PGM2L1	1.171	4.083	1.668E-04	1.015E-02	Up in PANC1-MRP4sh
ZC4H2	1.167	4.108	2.457E-04	1.355E-02	Up in PANC1-MRP4sh
NRGN	1.155	4.736	1.608E-04	9.839E-03	Up in PANC1-MRP4sh
TKT	1.147	9.726	1.236E-07	2.334E-05	Up in PANC1-MRP4sh
MYL9	1.139	6.382	8.255E-06	8.837E-04	Up in PANC1-MRP4sh
PREX1	1.133	7.482	4.961E-07	7.679E-05	Up in PANC1-MRP4sh
BDH1	1.130	3.996	7.706E-04	3.345E-02	Up in PANC1-MRP4sh
DCBLD2	1.128	6.084	1.212E-04	8.067E-03	Up in PANC1-MRP4sh
TSPYL5	1.109	4.466	6.436E-05	4.981E-03	Up in PANC1-MRP4sh
DSG2	1.105	5.799	5.224E-06	5.785E-04	Up in PANC1-MRP4sh
TPM1	1.089	8.241	1.502E-04	9.459E-03	Up in PANC1-MRP4sh
LPCAT1	1.084	8.967	7.044E-08	1.508E-05	Up in PANC1-MRP4sh
SAMD8	1.083	4.542	1.007E-04	7.145E-03	Up in PANC1-MRP4sh
GPHN	1.083	4.199	3.453E-04	1.760E-02	Up in PANC1-MRP4sh
CALD1	1.063	5.653	4.704E-04	2.272E-02	Up in PANC1-MRP4sh
PDLIM1	1.058	8.889	8.773E-08	1.761E-05	Up in PANC1-MRP4sh
FAM43A	1.031	6.558	1.932E-05	1.812E-03	Up in PANC1-MRP4sh
ELFN2	1.030	5.002	3.003E-04	1.588E-02	Up in PANC1-MRP4sh
FTSJ3	1.027	6.292	2.630E-06	3.112E-04	Up in PANC1-MRP4sh
MEF2C	1.026	4.609	1.414E-04	8.993E-03	Up in PANC1-MRP4sh
RASD1	1.026	4.979	3.093E-04	1.629E-02	Up in PANC1-MRP4sh
GALNT16	1.024	4.209	1.283E-03	4.847E-02	Up in PANC1-MRP4sh
ETHE1	1.024	4.179	8.471E-04	3.592E-02	Up in PANC1-MRP4sh
COL13A1	1.023	5.423	1.596E-04	9.812E-03	Up in PANC1-MRP4sh
CNOT11	1.019	5.928	2.069E-06	2.532E-04	Up in PANC1-MRP4sh
PORCN	1.002	4.006	1.255E-03	4.785E-02	Up in PANC1-MRP4sh

Supplementary Table 3. Top 10 down- and upregulated transcripts in PANC1-MRP4sh cells compared to PANC1-scramble cells

Top-**10 downregulated transcripts**			
**Gene symbol**	**Associated function**	**logFC**	**PValue**
COL4A5	Collagen fiber/focal adhesion. Extracellular matrix structural constituent. ERK signaling	-7.486	8.421E-04
GATA5	Cardiovascular development. Tumor suppressor in colon cancer	-5.263	3.044E-05
DSP	Organization of desmosomal cadherin-plakoglobin complexes. Maintain gap junctions	-4.611	3.888E-04
C20orf166-AS1	Antisense long non-coding RNA sequence	-4.210	8.538E-02
KRT19	Cytoskeleton regulation. Breast cancer. p38 signaling	-4.164	5.304E-11
LIMCH1	Stabilizes focal adhesions. Actin stress fibers-associated protein	-3.980	1.311E-03
COL6A2	Focal adhesions. ERK signaling	-3.963	1.128E-04
ADGRB1	DNA damage response. p53 signaling. GPCR activity	-3.842	1.263E-03
ELFN1	Granular cell carcinoma. Postsynaptic protein	-3.748	1.142E-22

Top-**10 upregulated transcripts**			
**Gene symbol**	**Associated function**	**logFC**	**PValue**
F13A1	Coagulation factor. Formation of fibrin clot	6.670	3.134E-23
PRICKLE2-AS1	Antisense sequence. Wnt signaling	6.228	5.879E-02
PRICKLE2	β-catenin-dependent Wnt signaling	5.425	4.732E-06
PLPP4	Actin dynamics during phagocytosis. Phospholipid metabolism	4.899	9.304E-04
RASL10A	GTPase activity. Neural tumor suppressor: inhibits cell growth and promotes caspase-independent cell death	4.260	6.936E-31
NMBR	Regulation of cell growth in lung and gastric cancer. GPCR signaling	4.138	1.544E-04
TRIP6	Focal adhesion. Actin stress fibers. Cell migration	4.084	6.343E-15
ZDHHC15	Palmitoyltransferase family. (*) Neuronal development and synaptic activity	4.008	3.413E-04
SUSD4	Inhibits the classical and alternative complement pathways	3.920	1.087E-05
MN1	Osteoblast maturation and function. Tumor suppressor	3.881	5.120E-11

Differential expression was established according to the Log Fold Changes. The associated function for each transcript was obtained from Genecards https://www.genecards.org/ or (*) Pub Med https://pubmed.ncbi.nlm.nih.gov/22155432/
